# Selective Removal of Contaminant Compounds from Polyol-Rich Fermented Broth by Multicomponent Adsorption on New Adsorbent Materials

**DOI:** 10.1021/acsomega.5c09201

**Published:** 2025-11-27

**Authors:** Danielle Garcia Ribeiro Galvão, Jan Galvão Gomes, Maria Eduarda Rampin de Almeida, Marcus Bruno Soares Forte

**Affiliations:** Bioprocess and Metabolic Engineering Laboratory (LEMeB), Food Engineering and Technology Department (DETA), 28132 Faculty of Food Engineering (FEA), University of Campinas (UNICAMP), R. Monteiro Lobato, 80, Campinas, São Paulo 13083-862, Brazil

## Abstract

Cocoa pod husk (CPH), a lignocellulosic agroindustrial byproduct, offers a sustainable source for producing high-value sugar alcohols such as arabitol and xylitol through microbial fermentation. However, the fermented broth contains a complex mixture of impurities, residual sugars, and phenolic compounds that impair polyol purity and require selective removal. This study investigated six adsorbent materials with distinct physicochemical properties for their ability to selectively purify polyol-rich broth via multicomponent adsorption. Activated carbons (acidic, basic, and neutral), a synthetic resin (Sepabeads SP700), and two ion-exchange resins (Diaion HPA512L and UBK550) were evaluated. pH variation (3–9) showed negligible influence on adsorption, allowing neutral conditions (pH 7) for subsequent tests. Among the materials, acid-activated carbon and HPA512L resin demonstrated superior clarification performance while preserving the polyol content. Kinetic studies fitted the pseudo-second-order model (*R* ^2^ = 0.93–1.00), indicating chemisorption. Adsorption equilibrium data were best described by Extended and Modified Langmuir isotherms (*R* ^2^ > 0.991), evidencing competitive adsorption between polyols. Acid-activated carbon showed the highest adsorption capacities, while the HPA512L resin offered operational benefits at elevated temperatures. These findings provide a foundation for designing efficient and sustainable downstream processes for polyol purification from biomass hydrolysates.

## Graphical abstract

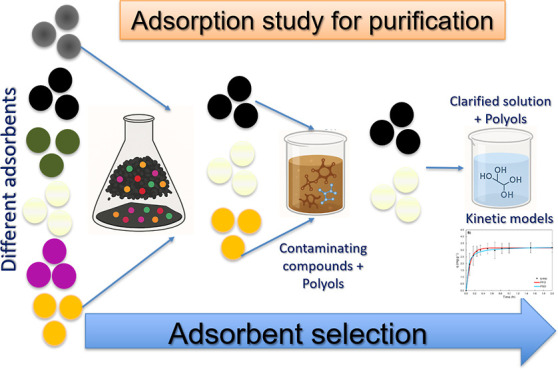

## Introduction

Cocoa pod husk biomass (CPH), a byproduct generated in large quantities during the processing of cocoa beans, represents a promising source of lignocellulosic materials.^1^ Globally, cocoa is widely cultivated for the production of chocolate and derivatives, reaching 4.84 million tons expected for the year 2024/2025.^2^ In Brazil, the sixth largest producer in the world, cocoa production grew significantly, reaching more than 296 thousand tons in 2023, representing a value of more than 800 million dollars, an increase of 12.69% compared to the previous year.^3^

Without proper use, the indiscriminate disposal of CPH can lead to soil contamination and the proliferation of pests, which directly affect the cultivation of this fruit.^1^ Although often underutilized, this biomass contains a series of components of industrial interest, such as cellulose, hemicellulose, and phenolic compounds, making it a potential raw material for several biotechnological applications.^4^ The valorization of CPH represents not only an opportunity to add value to agricultural residues but also a concrete strategy to promote more sustainable practices in the agrifood sector. This aligns with the principles of the circular economy and biorefinery and contributes directly to multiple United Nations Sustainable Development Goals (SDGs), particularly SDG 9 (Industry, Innovation and Infrastructure), SDG 12 (Responsible Consumption and Production), and SDG 13 (Climate Action). Therefore, this matrix can drive sustainability, support waste valorization, and reduce environmental impacts associated with agroindustrial processes.^5^

The hemicellulose fraction of CPH can be used to produce polyols of great industrial interest such as arabitol and xylitol. This process can occur biotechnologically through pentose fermenting microorganisms.^6,7^ The polyols arabitol and xylitol are widely studied compounds due to their functional properties and industrial applications. Both are low glycemic index sweeteners and are promising alternatives to sucrose, especially for consumers with dietary restrictions such as diabetics. In addition, they have prebiotic properties and can stimulate the growth of beneficial bacteria in the intestinal tract. Xylitol, in particular, is recognized for its noncariogenic property and is a common ingredient in dental products, such as chewing gum and toothpastes.^8^ Arabitol, although less commercially explored, has potential for applications in food and cosmetics due to its stability and moisturizing power.^9^ Thus, the valorization of these polyols from renewable sources, such as cocoa pod husk hydrolysate, represents a sustainable approach for the production of high added value ingredients.

In addition to the polyols of interest, cocoa pod husk hydrolysate contains a variety of other compounds such as residual sugars, organic acids, proteins, and phenolic compounds that produce pigments, the latter in large quantities.^10^ The presence of these compounds directly interferes with the purity of polyols and can compromise their application in sectors such as the food and pharmaceutical industries. In addition, some of these components can compete with polyols during separation processes, making selective recovery difficult and increasing purification costs.^11,12^ Therefore, the efficient removal of these interferents is essential to obtain products with a high degree of purity and commercial viability.

Adsorption has emerged as an effective strategy for the purification of biocompounds due to its selectivity, operational simplicity, and lower environmental impact compared to other techniques.^10,11,13–16^ In multicomponent systems, such as fermented broth, the interaction between different molecules and the adsorbent can significantly influence the efficiency of the process.^17,18^ Competition between compounds for the same active site and synergistic or antagonistic effects between solutes are determining factors in adsorption performance.^19^ Thus, understanding the behavior of polyols and interfering compounds under different conditions is essential for the development of optimized purification processes.

Previous studies have mainly focused on single-component adsorption systems or simplified synthetic solutions that do not adequately reflect the complexity of real fermentation broths. For instance, Choy et al.^20^ used extended Langmuir models to describe competitive adsorption of dyes from multicomponent effluents but not in biologically relevant broths. Other works (e.g., systematic reviews of heavy-metal adsorption) emphasize how coadsorbates significantly alter uptake capacities and selectivity.^21^ In bioprocess contexts, scarce studies address the adsorption of target biochemicals directly from whole fermentation broths; exceptions include the selective recovery of 2,3-butanediol from corn stover fermentation broth using nano-MFI zeolites and biobutanol recovery in ABE broths, both of which demonstrate that competitive components dramatically affect separation performance.^22,23^

The choice of adsorbent plays a critical role in the efficiency of polyol purification, influencing the selectivity and the ability to remove interfering compounds.^10^ Different classes of materials have been investigated for this purpose, including ion-exchange resins, activated carbon, and zeolites.^11,17^ Ion-exchange resins, for example, have a high affinity for certain polar compounds and can be functionalized to improve the selectivity. Activated carbon stands out for its large surface area and hydrophobic interactions, while zeolites offer porous structures that favor selective separation processes. The evaluation of the performance of these adsorbents in multicomponent systems allows for the definition of the best operational conditions for the efficient recovery of the polyols of interest.

In this context, different types of adsorbents with different physical and chemical characteristics were selected and evaluated, with the aim of promoting the selective removal of undesirable compounds present in the fermented broth and thus enabling the purification of the polyols arabitol and xylitol. The materials used included activated carbons with different surface characteristics, which allow the exploration of electrostatic and hydrophobic interactions, in addition to the synthetic adsorbent SP700, known for its porous polymeric structure and high adsorption capacity in aqueous solutions. In addition, the ion-exchange resins HPA512L and UBK550 were used, which act based on selective ion-exchange mechanisms, especially effective for polar compounds. The diversity of these materials allowed a comprehensive comparative analysis, considering not only the efficiency of contaminant removal but also the competitive behavior between the components of the multicomponent system, contributing to the development of a more efficient and sustainable purification process. To the best of our knowledge, this is the first study to apply multicomponent adsorption modeling (Extended and Modified Langmuir) to the purification of polyols from real broth conditions, integrating kinetic and equilibrium analyses in a single framework. This novelty directly addresses the limitations of earlier studies and provides new insights into the separation of structurally similar compounds (arabitol and xylitol) in competitive systems.

## Materials and Methods

### Chemicals and Model Solution

Xylitol, L-arabitol, xylose, and arabinose (analytical grade) were purchased from Sigma-Aldrich. Alkaline lignin (used as a model for colored compounds) was purchased from Sigma-Aldrich. Ultrapure water was used in all of the preparations. The colored compounds were read at absorbances of 420 and 560 nm. An analytical curve with known concentrations was used to measure the concentration of these compounds.

### Adsorbents and Pretreatment

The adsorbents evaluated were acidic (AC-A), basic (AC-B), and neutral activated carbon (AC-N) (Clarimex, Brazil), the polymeric adsorbent Sepabeads SP700 (Mitsubishi Chemical), and the ion-exchange resins Diaion HPA512L and UBK550 (Mitsubishi Chemical). Activated carbons were milled (Tecnal TE-631), sieved to 40–100 mesh (0.420–0.149 mm), and dried at 60 °C prior to use. Resins were used in the wet (as-supplied) form; SP700 was conditioned by sequential washing with water and methanol according to manufacturer recommendations, and resins were rinsed with deionized water before experiments.^17,24,25^ Manufacturer data sheets were used to compile the physicochemical properties presented in Table 1; additional parameters of BET surface area, pore volume, and mean pore diameter were obtained when they were available from suppliers or from our characterization.

**Table 1:** Physicochemical Characteristics of Some Commercial Adsorbents Used in the First Stage: Adsorbent Screening^a^ ^b^

grade name	matrix	surface area (m^2^·g^–1^)	pore radius (Å)	water content (%)	particle size distribution (mm)	density (g·mL^–1^)
Sepabeads SP700	DVB-EVB	1100	90	60–70	0.25–0.70	1.02
Diaion HPA512L	St-DVB	9	191	63–73	0.425–1.18	1.05
Diaion UBK550	St-DVB	-	-	46.0–49.5	0.2–0.24	1.28

a Product description obtained from www.diaion.com (Website accessed 12-05-2025).

b Data presented according to the manufacturer’s Product Data Sheet, where DVB is divinylbenzene, EVB is ethylvinylbenzene, and St is styrene.

### Initial Screening—Real Fermented Broth

An initial screening employing six distinct adsorbent materials was conducted to assess their efficacy in clarifying a real fermented broth and to facilitate the preliminary selection of suitable adsorbents. For this evaluation, a fermented broth containing 10 g·L^–1^ xylitol, used as a reference concentration, served as the adsorbate solution. The broth, comprising polyols and pigmented compounds, was tested at three pH levels (3, 7, and 9), which were adjusted using buffer solutions of H_2_SO_4_ and NaOH.

Adsorption experiments were carried out in batch mode using thermoblocks (Loccus DBH-S), under controlled conditions: temperature at 30 °C, agitation at 1500 rpm, solution volume of 1.2 mL, adsorbent concentration of 50 g·L^–1^, and a contact time of 4 h. Sampling was performed in duplicate using sacrificial aliquots. The percentage reduction of each target component, calculated according to eq 1, was employed as the criterion for adsorbent selection.

1 $$\%Reduction=\frac{C_{0}-C_{e}}{C_{0}}\times 100$$

where *C*_0_ and *C*_e_ are the initial and equilibrium concentrations (mg·L^–1^), respectively. Results from the screening are reported as the average of the duplicate sacrificial samples.

### Model Solution for Kinetic and Equilibrium Studies

For kinetic and equilibrium experiments, a defined model solution was used, prepared to simulate the main components relevant to the fermented broth studied in this work. The model composition used in the experiments reported in this manuscript was xylitol 10 g·L^–1^, L-arabitol 5 g·L^–1^, arabinose 2 g·L^–1^, xylose 2 g·L^–1^, and lignin 2 g·L^–1^. Solutions were prepared by dissolving the required amounts in ultrapure water and used fresh.

### Kinetic Adsorption Study

Kinetic adsorption experiments were conducted to identify the optimal operational parameters for subsequent equilibrium studies. The assays were performed in 250 mL Erlenmeyer flasks containing 100 mL of the model solution and an adsorbent concentration of 50 g·L^–1^. The flasks were maintained in an orbital shaker at 200 rpm, under controlled temperatures of 30 and 50 °C, for a duration of up to 48 h. All experiments were carried out in triplicate with aliquots collected at predetermined time intervals. No pH adjustment was required. Each aliquot represented a discrete experimental data point, and component concentrations were determined according to the procedures described in the analytical methods section. The adsorption capacity at time *t* was calculated as

2 $$q_{t}=\frac{\left(C_{0}-C_{t}\right)\cdot V}{m}$$

where *q*_*t*_ is the adsorption capacity at time *t* (mg·g^–1^), *C*_0_ and *C*_*t*_ are initial and time-*t* concentrations (mg·L^–1^), *V* is the solution volume (*L*), and *m* is the mass of adsorbent (*g*).

The experimental kinetic data were fitted to the pseudo-first-order (PFO—eq 3) and pseudo-second-order (PSO—eq 4) models, as originally described by Lagergren and by Ho and McKay,^26,27^ respectively,

3 $$\frac{{dq}_{t}}{dt}=k_{1}\left(q_{e}-q_{t}\right)$$

4 $$\frac{{dq}_{t}}{dt}=k_{2}{\left(q_{e}-q_{t}\right)}^{2}$$

where *t* is the time (h), *q*_e_ and *q*_*t*_ are the adsorption capacity at equilibrium and at time *t* (mg·g^–1^), *k*_1_ is the PFO rate constant (h^–1^), and *k*_2_ is the PSO rate constant (g·mg^–1^·h^–1^). Fitting was performed using Statistica software; reported kinetic parameters are averages (±standard deviation) of triplicate experiments.

Note on sampling density: the experiments and sampling times reported in this manuscript follow the protocol described above. No additional rapid-initial-stage experiments (e.g., extra points at 1–4 min) were performed beyond the time points actually collected; the lack of very early time points is discussed in the manuscript as a limitation for fitting extremely fast adsorption events.

### Adsorption Equilibrium Isotherms (Multicomponent)

The adsorption equilibrium of interfering compounds, specifically colored substances derived from lignin, xylose, and arabinose, and polyols (arabitol and xylitol), was investigated under two temperature conditions (30 and 50 °C), utilizing the same experimental setup employed in the kinetic studies. The initial concentrations of adsorbates, contact time, temperature, and adsorbent mass were defined based on the outcomes of the kinetic experiments.

The equilibrium adsorption capacity (*q*_e_) was determined using eq 5:

5 $$q_{e}=\frac{\left(C_{0}-C_{e}\right)\cdot V}{m}$$

where *C*_*i*_ and *C*_e_ represent the initial and equilibrium concentrations of the adsorbates (g·L^–1^), respectively; *V* is the volume of the solution (*L*); and *m* denotes the mass of the adsorbent (*g*).

Among the various models used to describe adsorption phenomena, the Langmuir isotherm remains one of the most widely applied. This model is based on several key assumptions: a finite number of adsorption sites, uniform energy distribution across these sites, absence of interactions between adsorbed molecules, monolayer adsorption, and occupancy of each site by a single molecule.

For systems involving multicomponent adsorption, several extended models have been proposed to account for the competitive interactions among solutes. The Extended Langmuir (EL) model (eq 6) assumes that all components compete for the same adsorption sites and share the saturation capacity, thereby serving as a direct generalization of the original Langmuir formulation. The Modified Competitive Langmuir (MCL) model (eq 7) incorporates interaction parameters to more accurately represent competitive adsorption behavior among different solutes. Conversely, the Noncompetitive Langmuir (NL) model (eq 8) posits that each component adsorbs independently, without direct competition for adsorption sites. Lastly, the Langmuir–Freundlich (Sips) model (eq 9) integrates elements of both Langmuir and Freundlich isotherms, accommodating surface heterogeneity and variable adsorption intensities, thus offering enhanced flexibility for fitting experimental data from heterogeneous systems.

6 $$q_{i}=q_{\max,i}\frac{k_{i}C_{i}}{1+\sum_{j=1}^{n}k_{j}C_{j}}$$

7 $$q_{i}=q_{\max}\frac{k_{i}C_{i}}{1+\sum_{j=1}^{n}k_{j}C_{j}}$$

8 $$q_{i}=q_{\max,i}\frac{k_{i}C_{i}}{1+k_{i}C_{i}}$$

9 $$q_{i}=q_{\max,i}\frac{{\left(k_{i}C_{i}\right)}^{ni}}{1+\sum_{j=1}^{n}{\left(k_{j}C_{j}\right)}^{nj}}$$

where *q*_e_ (mg g^–1^) is the amount of adsorbate adsorbed at equilibrium, *q*_max_ (mg g^–1^) is the maximum amount of adsorbate adsorbed, *k*_i_ (L mg^–1^) and C_i_ is the concentration on equilibrium.

### Model Evaluation

The coefficient of determination (*R* ^2^) (eq 10) and the average absolute deviation (%D) (eq 11) were employed to assess the goodness of fit of the kinetic, diffusion, and isotherm models to the experimental data.

10 $$R^{2}=1-\frac{\sum_{i=1}^{n}{\left(q_{i}-\mathrm{q̂}\right)}^{2}}{\sum_{i=1}^{n}{\left(q_{i}-\mathrm{q̅}\right)}^{2}}$$

11 $$\%D=\left(\frac{1}{n}\left|\sum_{i=1}^{n}\frac{q_{i}-{\mathrm{q̂}}_{i}}{q_{i}}\right|\right)\times 100$$

In these equations, *n* denotes the number of experimental data points, *q*_i_ is the observed adsorption capacity (mg g^–1^), $\hat{q_{i}\;}$ is the mean of the observed adsorption capacities (mg g^–1^), and *q̅* represents the predicted adsorption capacity (mg g^–1^). These statistical metrics provide quantitative measures of the model accuracy and predictive performance.

### Analytical Methods and Characterization

Quantification of sugars was performed using High Performance Liquid Chromatography (HPLC) (Accela, Thermo Fisher Scientific), equipped with a refractive index (RI) detector and an HPX-87H column. Chromatographic conditions included a column temperature of 35 °C, a mobile phase of 0.01 N sulfuric acid, and a flow rate of 0.6 mL min^–1^, following a methodology adapted from ref 28. The polyols arabitol and xylitol were quantified via HPLC with infrared detection (HPLC-IR), employing a Hi-Plex Ca column operated at 65 °C, using ultrapure water as the mobile phase at a flow rate of 0.58 mL min^–1^, according to a protocol adapted from ref 6.

Colored compounds were assessed by measuring absorbance using a spectrophotometer at wavelengths of 420 and 560 nm, in accordance with the guidelines established by the International Commission for the Unification of the Methods of Sugar Analysis (ICUMSA).^10,11^ Absorbance at 420 nm was specifically used to monitor chromophores associated with phenolic compounds. Concentrations were determined from calibration curves when available and are expressed in relative absorbance units.

Selected adsorbents were characterized by Fourier Transform Infrared Spectroscopy (FTIR). For FTIR analysis, pellets were prepared using the potassium bromide dilution method, consisting of a 1% sample and 99% KBr. Spectral data were acquired using a Shimadzu IRPrestige-21 spectrophotometer (Shimadzu, Kyoto, Japan) at ambient temperature (25 °C), with a scanning range of 400–4000 nm and a resolution of 4 cm^–1^. Surface morphology analysis was conducted using a Hitachi TM400Plus benchtop scanning electron microscope (SEM).

## Results and Discussion

### Initial Selection of Adsorbents

The initial screening of the adsorbents was performed using the actual fermented broth obtained from the lignocellulosic material. This broth contained the target polyols (xylitol and arabitol) together with residual sugars, organic acids, and colored phenolic compounds derived from lignin, which are undesirable in the downstream process. The purpose of this step was to identify the materials adsorbents capable of clarifying the broth by selectively removing colored compound contaminants while minimizing the loss of polyols.

The initial pH of the fermented broth was 5.5 ± 0.2, corresponding to its natural condition after fermentation, and no pH adjustment was made during the clarification tests. This choice allowed for the evaluation of adsorbent performance under realistic process conditions. Preliminary trials showed that pH modification led to slightly higher pigment removal but also to significant polyol losses; therefore, maintaining the natural pH was considered to be a more sustainable and representative condition for process integration.

Batch experiments were conducted under standardized conditions (30 °C, 4 h contact time, 50 gL^–1^ adsorbent dosage, and 1500 rpm agitation). The coals were dried at 60 °C for 12 h before use in order to remove impurities and moisture, and the resins were used according to the manufacturer. Regeneration was not applied at this stage, since the purpose was to compare pristine materials. Each condition was tested in duplicate using sacrificial samples, and the results are reported as the mean values. The removal percentages were calculated from the initial and final concentrations determined by HPLC and UV–vis analyses.

The results of the screening are summarized in Table 2. A clear variation in adsorption performance was observed among the materials, indicating that the surface chemistry strongly influenced the interactions with the compounds present in the broth. Among the activated carbons tested, the one with an acidic surface exhibited the highest color removal efficiencies at wavelengths of 420 and 560 nm while maintaining relatively low adsorption of arabitol and xylitol. This selective adsorption is desirable for purification purposes as it enhances the removal of unwanted compounds without significantly affecting valuable sugar alcohols.

**Table 2:** Percentage Reduction after Adsorption with Adsorbents and Different pH^a^

average % reduction^b^							
	pH	xylose	arabinose	xylitol	arabitol	Abs 420 nm	Abs 560 nm
(AC-A)	3	30.17 ± 1.39	11.07 ± 0.08	7.85 ± 0.91	0.47 ± 0.23	76.88 ± 0.57	31.56 ± 0.62
	7	35.92 ± 4.06	10.07 ± 3.46	6.84 ± 5.37	6.56 ± 0.46	80.64 ± 1.23	37.60 ± 2.81
	9	29.52 ± 4.22	11.40 ± 4.69	8.94 ± 0.00	10.33 ± 2.15	79.59 ± 0.73	25.69 ± 4.11
(AC-B)	3	31.59 ± 3.70	17.94 ± 1.00	16.02 ± 1.59	15.09 ± 4.45	69.78 ± 1.22	24.51 ± 3.95
	7	40.74 ± 5.59	14.68 ± 5.63	12.96 ± 4.80	21.31 ± 4.17	69.92 ± 3.51	40.51 ± 6.26
	9	23.58 ± 5.86	5.15 ± 5.43	9.09 ± 0.00	12.77 ± 3.01	79.24 ± 2.95	24.96 ± 0.06
(AC-N)	3	25.38 ± 2.00	10.96 ± 1.47	8.84 ± 0.87	10.69 ± 1.78	75.26 ± 3.48	33.62 ± 0.25
	7	31.15 ± 4.79	8.42 ± 4.83	10.14 ± 4.00	22.66 ± 1.91	45.00 ± 3.98	51.34 ± 4.78
	9	29.38 ± 0.00	10.06 ± 0.56	8.93 ± 1.27	4.43 ± 0.68	55.94 ± 9.21	25.36 ± 4.19
HPA 512L	3	20.37 ± 1.69	15.16 ± 1.39	13.17 ± 1.47	4.72 ± 5.78	73.77 ± 5.14	35.75 ± 4.54
	7	14.56 ± 1.76	10.35 ± 0.97	9.63 ± 1.77	1.82 ± 1.29	84.57 ± 4.24	89.17 ± 2.93
	9	6.75 ± 4.19	7.19 ± 1.91	7.30 ± 1.48	1.00 ± 0.86	79.75 ± 2.15	70.70 ± 3.28
SP700	3	22.00 ± 1.23	14.34 ± 0.23	11.19 ± 0.76	5.66 ± 0.63	81.85 ± 6.34	78.44 ± 4.01
	7	20.54 ± 1.28	12.57 ± 0.88	10.83 ± 1.13	5.92 ± 2.32	78.87 ± 0.92	47.20 ± 5.89
	9	15.52 ± 2.18	10.90 ± 3.50	9.77 ± 1.46	7.29 ± 4.73	78.72 ± 0.84	44.10 ± 1.72
UBK550	3	5.34 ± 2.62	4.42 ± 1.93	3.89 ± 1.67	0.10 ± 2.22	68.93 ± 0.25	68.82 ± 0.06
	7	2.93 ± 2.23	3.52 ± 0.97	4.28 ± 0.77	0.33 ± 0.46	37.54 ± 2.26	25.92 ± 6.26
	9	4.03 ± 1.39	0.83 ± 0.10	3.26 ± 0.20	5.47 ± 3.87	19.45 ± 3.58	19.51 ± 0.67

a Where AC-A is the activated acid carbon, AC-B is the activated basic carbon, and AC-N is the activated neutral carbon.

b The values represent the mean ± standard deviation of independent experimental replicates. The relatively larger deviations observed for some samples reflect the variability inherent in the spectrophotometric analysis of multicomponent systems, particularly when using fermented broths with complex composition. These variations are within the expected experimental range, and it is possible to clearly identify which adsorbents caused greater clarification of the system.

This superior performance is attributed to the presence of acidic functional groups on the carbon surface, which impart an overall acidic character to the material and promote the adsorption of basic compounds present in the medium. Huang et al.^29^ demonstrated that acid activation of charcoal enhances its ammonia adsorption capacity due to the formation of Brønsted acid sites. These sites interact with ammonium ions (NH_4_^+^), as evidenced by FTIR analyses in their study, which revealed N–H and O–H bonds indicative of NH_4_^+^ formation and Brønsted acid-type interactions. Although these findings are not from the present work, they provide a useful framework for interpreting the behavior of acid-activated carbon in our system. The fermented broth may contain peptide fragments derived from fermentation supplements, as well as phenolic compounds originating from lignin degradation in the biomass.^7^ Phenolic compounds, such as phenol, can interact with activated carbon through the donor–acceptor complex formation. Specifically, electron-donating groups (e.g., carbonyls) on the carbon surface may engage with the aromatic ring of phenol, which acts as an electron acceptor. Additionally, hydroxyl groups on the carbon surface may facilitate adsorption through hydrogen bonding mechanisms.^30,31^

In contrast, basic and neutral activated carbons exhibited lower removal efficiencies for colored compounds while promoting a higher adsorption of the target polyols. This behavior suggests that surface charge interactions favor the binding of polyols over the compounds responsible for color in the fermented broth, indicating reduced affinity for chromophore species under neutral or basic surface conditions. In turn, the UBK550 resin also showed limited effectiveness among the resins under the same conditions. The poor performance of UBK550 may be associated with its quaternary ammonium functional groups, which have a low layer of neutral and weakly acidic components, typically present in fermentation broth.^6^

The polymeric adsorbent SP700 showed intermediate performance, removing a moderate number of pigments but with partial loss of polyols. Regarding the highlighted adsorbent resins, Sepabeads SP700 and Diaion HPA512L may present distinct mechanisms of interaction with the fermented broth. Sepabeads SP700, a nonionic polystyrene-divinylbenzene resin, demonstrated efficiency in the removal of nonpolar and aromatic compounds through hydrophobic interactions between the aromatic rings of its matrix and phenolic or aromatic molecules that may be present in the fermented broth of lignocellulosic biomass.^32^ These interactions facilitate the adsorption of impurities responsible for coloring and other nonpolar substances, contributing to clarification. Although there are not many studies that used the Diaion HPA512L resin, its operating principle involves ion exchange, acting through electrostatic interactions in the removal of charged impurities.

According to Nakano and Betti,^33^ who employed resins from the same family as Diaion HPA512L (specifically HPA75 and HPA25L) to separate glycomacropeptide (GMP) from κ-casein with high purity, a result of great relevance to the food industry and human health, as GMP can serve as a marker of milk quality, these ion-exchange resins are effective in selectively removing ionizable compounds through electrostatic interactions. . Thus, depending on the pH of the solution, ionizable compounds, such as organic acids or phenolic ions present in the protein broth, interact with the charged functional groups of the resin through ion-exchange mechanisms. This selective adsorption of ionic species promotes the removal of polar contaminants and residual fermentation byproducts, improving the purity of the clarified solution.

Acidic activated carbon and HPA512L resin were identified as the most promising adsorbents for subsequent studies using a real fermented broth, with SP700 resin also selected due to its intermediate performance. These materials demonstrated an effective balance between high clarification efficiency and minimal loss of target polyols, highlighting their potential for downstream purification within an integrated biorefinery framework. Their selection was further supported by consistent performance across a range of pH values (3, 7, and 9), indicating chemical stability under varying conditions. As a result, pH 7 was chosen for the following experiments given its intermediate nature, chemical stability, and operational convenience. This approach is particularly relevant considering the intense coloration of fermented broths derived from cocoa pod husk, which is attributed to phenolic compounds and other impurities, whose removal is essential for applications in food and pharmaceutical sectors.

### Adsorbents Characterization

The adsorbent materials that showed the best performance in the preliminary screening, the acidic activated carbon, the cation-exchange resin HPA512L, and the nonionic resin SP700, were selected for physicochemical characterization before and after the adsorption experiments using the model polyol solution. This analysis aimed to elucidate the structural and chemical modifications that occurred on the surface of each material as a result of the adsorption process and to relate these changes to the observed adsorption mechanisms.

Fourier Transform Infrared (FTIR) spectroscopy (Figure 1) confirmed the presence of characteristic functional groups associated with each material. For the activated carbon (Figure 1A), broad absorption at ∼3440 cm^–1^ was attributed to O–H stretching from hydroxyl or carboxylic groups,^34^ while the bands between 1710 cm^–1^ and 1620 cm^–1^ corresponded to C=O stretching of carbonyl and carboxylate functionalities.^35,36^ These oxygenated groups confer surface acidity and enhance the interaction with basic or polar solutes in aqueous media. According to Iwanow and collaborators,^37^ the most characteristic bands for materials such as activated carbon are at ∼3500, 1700, and 1610 cm^–1^, as can be seen in Figure 1A. It is also observed that there is a decrease in the intensity of the band at ∼3440 cm^–1^, which indicates that the corresponding functional group was involved in the interaction with the adsorbate, which can be seen for activated carbon after adsorption. These results corroborate the efficiency of activated carbon in clarifying the medium.

**Figure 1:**
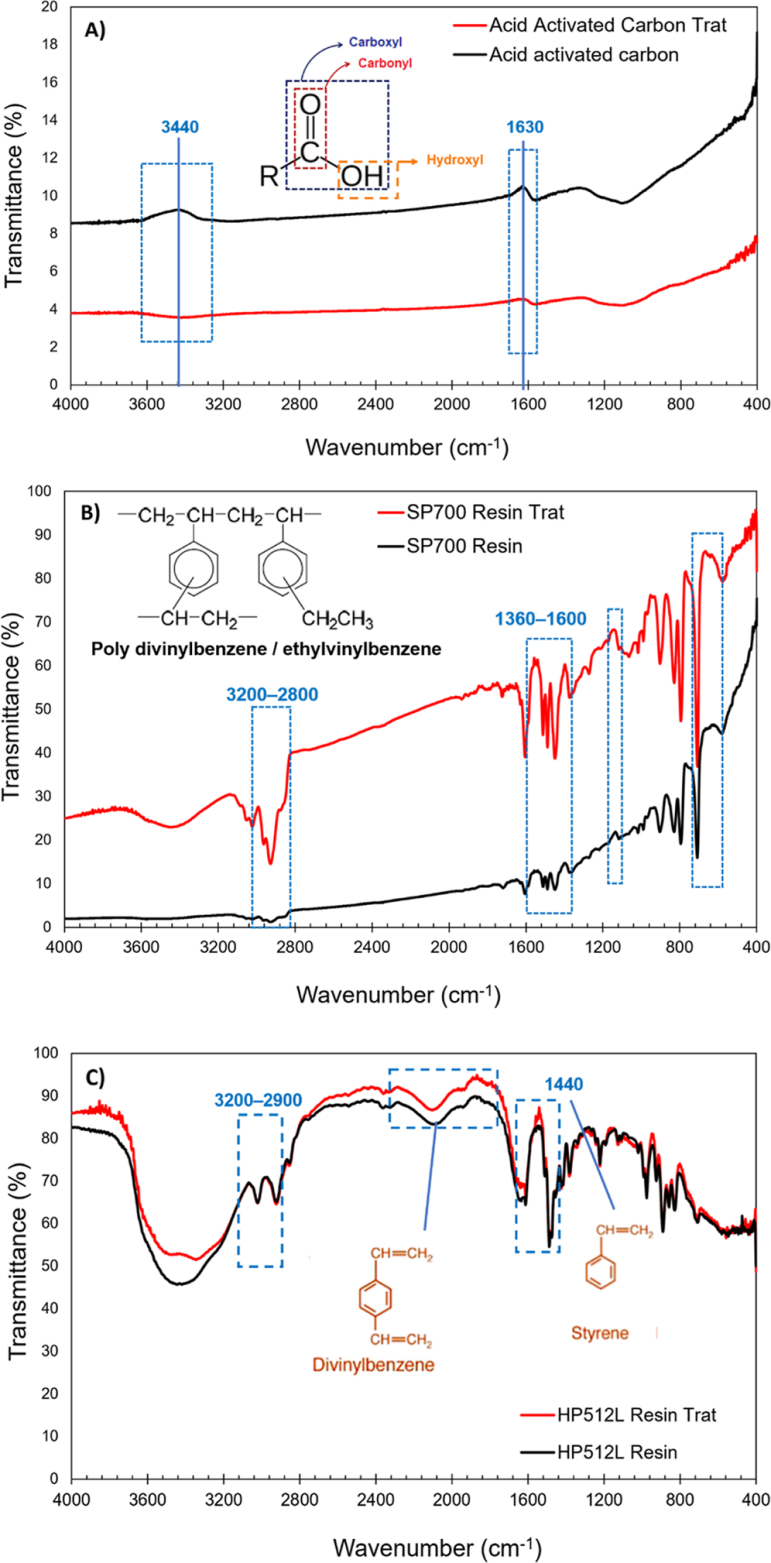
Trat is the absorbent after the adsorption process. FTIR spectra of the adsorbents before and after the adsorptive process with the model solution containing polyols and lignin. (A) is the acid-activated carbon; (B) is the SP 700 resin, and (C) is the HPA512L resin.

The FTIR spectra of the Sepabeads SP700 resin (Figure 1B), acquired before and after exposure to the model phenolic solution, revealed distinct spectral modifications indicative of molecular interactions at the polymer surface. Notably, the appearance and intensification of absorption bands in the region of 3200–2800 cm^1^ and 1360–1600 cm^1^ were observed, corresponding to the aromatic stretching and bending bands −CH2– and −CH–, present in materials whose matrix is a polystyrene-divinylbenzene polymer.^38^ These changes suggest the incorporation of aromatic species into the resin matrix. Furthermore, enhanced signals in the 1600–1500 cm^1^ range were attributed to C=C stretching of the aromatic rings, while the increased intensity between 1230 and 1030 cm^–1^ was associated with C–O–C stretching of the aryl ether (C–O–C) functionalities, commonly found in lignin-derived compounds. Collectively, these spectral changes provide compelling evidence for the adsorption of these compounds on the SP700 surface, likely mediated by hydrophobic interactions and π–π stacking between the aromatic moieties of the adsorbates and the styrenic backbone of the resin.^39^

Figure 1C presents the FTIR spectra of the Diaion HPA512L resin, a highly porous strong-base anion exchanger composed of a styrene-divinylbenzene (DVB) matrix functionalized with trimethylammonium groups. The band observed near 1400 cm^–1^ can be attributed to styrene (C–H bending vibrations),^40^ and at 1600 cm^–1^ to aromatic C=C stretching vibrations inherent to the styrenic structure, while the signal around 2360 cm^–1^ is tentatively associated with C–H stretching modes of aliphatic segments of the polymer matrix. The bands between 1940 and 1700 cm^–1^ and 1600 and 1400 cm^–1^ refer to the substituted bonds of the aromatic rings of divinylbenzene.^41^ Comparative analysis of the spectra before and after adsorption revealed pronounced changes in regions characteristic of lignin compounds, notably, around 3400 cm^–1^ (O–H stretching), 2900 cm^–1^ (C–H stretching), and 1600–1500 cm^–1^ (aromatic ring vibrations). The appearance or intensification of these bands in the postadsorption spectrum suggests an interaction of these groups with the resin surface. These spectral modifications support the hypothesis that the adsorption process facilitated the removal of chromophores compounds from the solution, contributing to their clarification through molecular interactions such as hydrogen bonds and π–π stacking between the aromatic portions of the adsorbates and the styrenic structure of the resin.

Figure 2 illustrates the surface morphology of the adsorbents before and after adsorption. As seen in Figure 2A, before sorption, the surface morphology of the activated carbon presents irregular cavities and fine open pores. A regular structure and developed pores can be observed after sorption in Figure 2B, which shows a smoother surface of activated carbon. The SP700 and HPA512L resins presented smooth, spherical particles before adsorption (Figure 2C,E), and after contact with the solution, their surfaces appeared less uniform, consistent with partial pore filling or surface coating by adsorbed solutes (Figure 2D,F). The insets also reveal that the macroscopic appearance of the adsorbent resins SP700 and HPA512L underwent a noticeable surface color change following adsorption, indicating the occurrence of adsorption on the resin surfaces.

**Figure 2:**
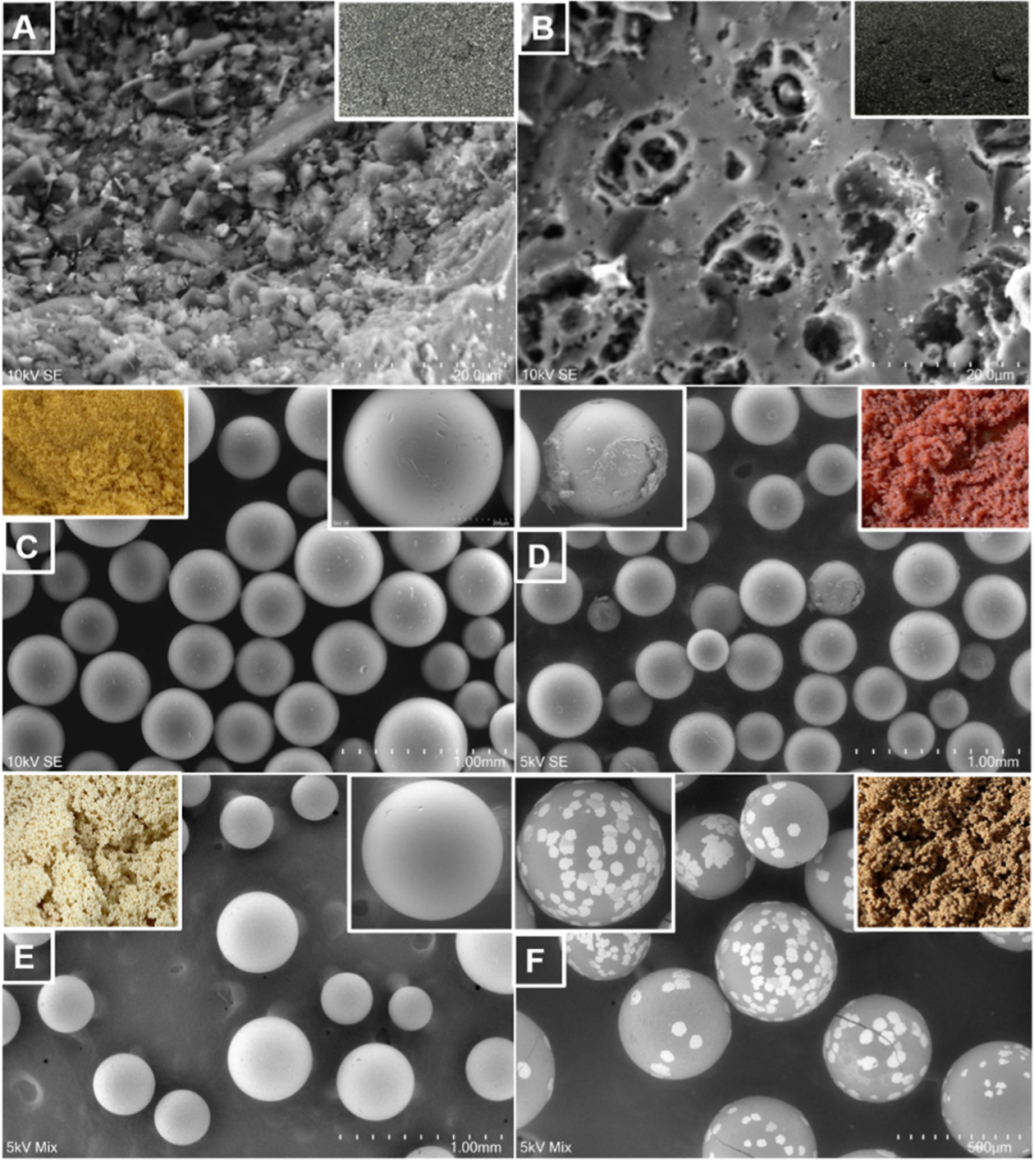
Scanning Electron Microscopy (SEM) images of the selected adsorbents before and after adsorption from the model solution, where (A) represents the acidic activated carbon before adsorption and (B) the same material after adsorption. (C) corresponds to the Sepabeads SP700 resin before adsorption and (D) the same resin after adsorption. (E) refers to the Diaion HPA512L resin before adsorption and (F) the same resin after adsorption. Insets show the macroscopic appearance and higher-magnification views of each adsorbent.

The textural properties of the three selected adsorbents: acidic activated carbon (AC-A), Sepabeads SP700, and Diaion HPA512L were determined using the Brunauer–Emmett–Teller (BET)^42^ and Barrett–Joyner–Halenda (BJH) methods.^43^ The obtained parameters, including specific surface area, total pore volume, and mean pore diameter, are summarized in Table 3, where both the supplier data “product description” and the experimental values obtained in this work are reported for direct comparison. Acidic activated carbon (AC-A) presented a BET surface area of 552.86 m^2^ g^–1^ and a total pore volume of 2.13 cm^3^ g^–1^. The mean pore diameter was 7.7 nm, confirming its mesoporous nature. The high surface area and moderate pore width result from the extensive network of fine pores generated during chemical activation, providing numerous accessible adsorption sites for the solute molecules.

**Table 3:** Physicochemical Properties of the Adsorbents Were Provided by the Supplier and Confirmed through Experimental Analysis^a^

adsorbent	BET surface area (m^2^·g^–1^)	total pore volume (cm^3^·g^–1^)		average pore diameter (Å)		pore type^b^		
	product description	this work	product description	this work	product description	this work	product description	this work
AC-A	–	552.86	–	2.13 ± < 0.00	–	76.94	–	mesopore
SP700	1100	1149.67	2.20	1.84 ± < 0.00	90	31.96	mesopore	mesopore
HPA512L	9.00	9.02	0.11	4.99 ± < 0.00	191	278.94	mesopore	mesopore

a Product descriptions were retrieved from the official Website (www.diaion.com; accessed on October 15, 2025).

b The classification for pore type was according to IUPAC.^44^

Sepabeads SP700 had the largest surface area among the adsorbents evaluated, at 1149.67 m^2^ g^–1^, consistent with the supplier’s specification (1100 m^2^ g^–1^). The total pore volume (2.20 cm^3^ g^–1^) and the average pore diameter of 3.2 nm also agree with the product description (3.1 nm), confirming its uniform mesoporous structure. The SP700 matrix, composed of nonionic polystyrene-divinylbenzene, provides a hydrophobic environment that promotes the adsorption of aromatic and nonpolar molecules through π–π and van der Waals interactions.

The Diaion HPA512L ion-exchange resin also presented a surface area (9.02 m^2^ g^–1^) very close to the supplier’s specification (9 m^2^ g^–1^) but a significantly larger pore diameter. The experimentally determined mean pore diameter was 27.89 nm (278.94 Å), placing it in the mesoporous range, while the product description indicates a slightly smaller value (19.1 nm). This difference may be related to the hydration state of the sample or partial collapse or swelling of the polymer matrix during nitrogen adsorption analysis.

### Adsorption Kinetic Studies

In this study, the adsorption kinetics were obtained for all six components present in the model solution: xylose, arabinose, xylitol, arabitol, and the compounds detected at 420 and 560 nm, using the three selected adsorbents, acid activated carbon (AC-A), Sepabeads SP700, and Diaion HPA512L, at two temperatures (30 and 50 °C). Adsorption over contact time was investigated over a 24 h period, with sampling at various intervals during the first two hours to better describe the initial phase of rapid adsorption, followed by a slower equilibration. The experiments were conducted in triplicate, and the mean values with standard deviations are presented in the kinetic graphs.

Kinetic adsorption studies were performed for the three adsorbents and are presented in Figures 3 and 4. Although all components exhibited some degree of adsorption, the adsorbents showed significantly higher affinity for the colored compounds, while the sugars and polyols showed lower adsorption, with removal rates of approximately 10% at 30 °C and between 10% and 20% at 50 °C.

**Figure 3:**
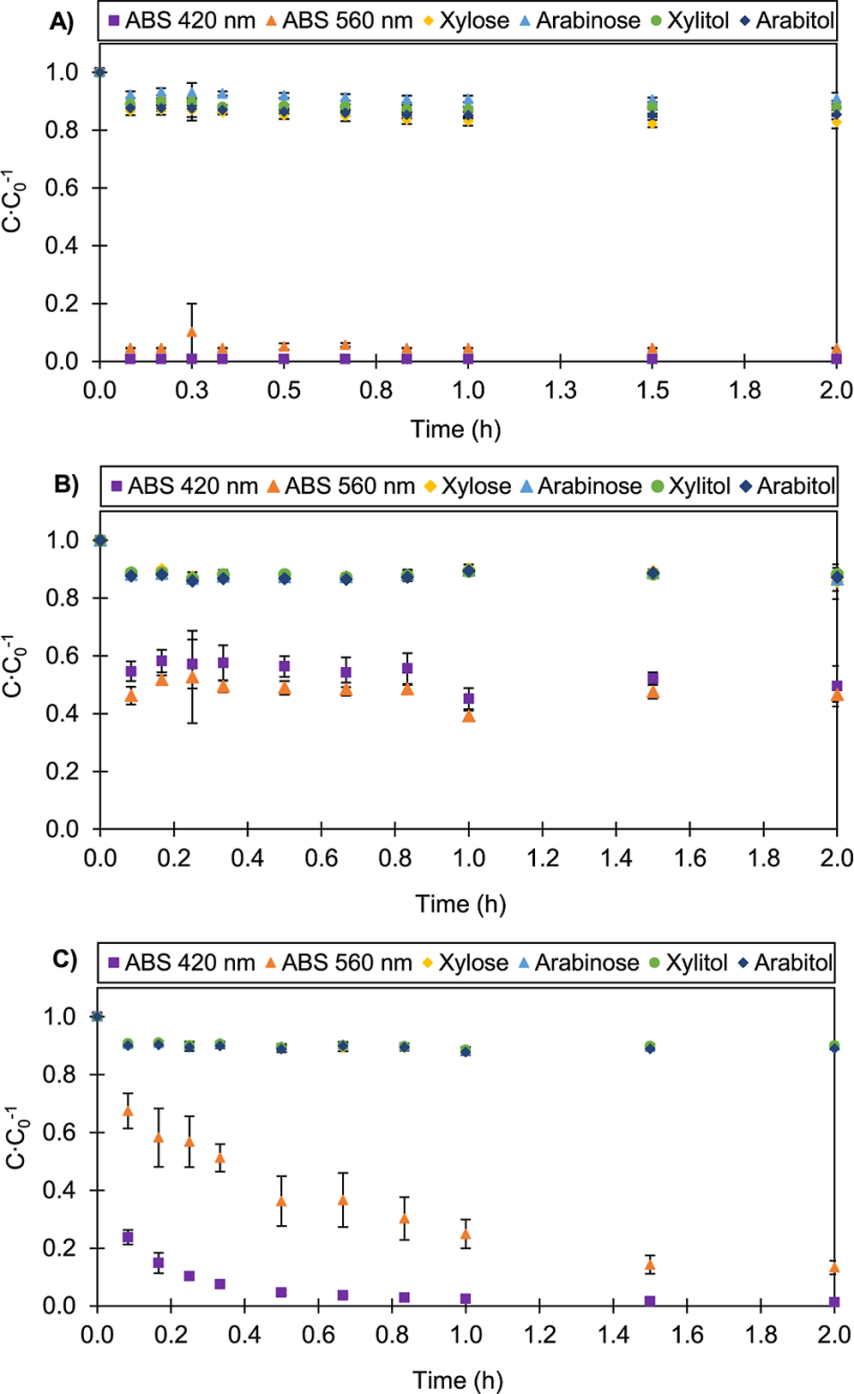
Adsorption kinetics of xylose, arabinose, l-arabitol, xylitol, and colored compounds on acid-activated carbon (AC-A) (A), resin SP700 (B), and HPA512L (C) at temperature 30 °C, an adsorbent content of 50 g·L^–1^, and a solution volume of 0.1 L.

**Figure 4:**
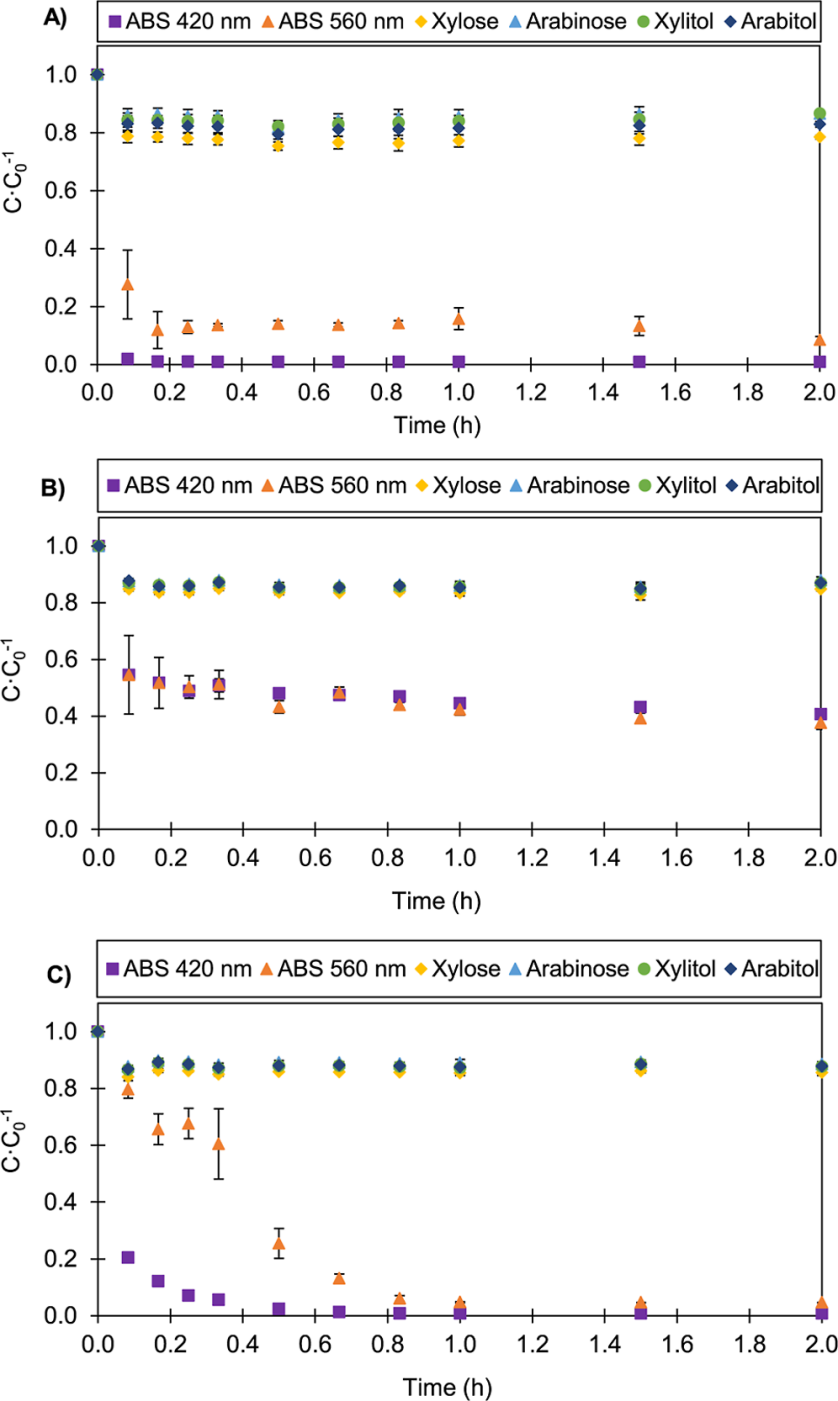
Adsorption kinetics of xylose, arabinose, l-arabitol, xylitol, and colored compounds on acid-activated carbon (AC-A) (A), resin SP700 (B), and HPA512L (C) at temperature 50 °C, an adsorbent content of 50 g·L^–1^, and a solution volume of 0.1 L.

Three distinct behaviors were observed at 30 °C (Figure 3) for the colored compounds: they were completely adsorbed by the acidic activated carbon at the initial sampling times; adsorption on the SP700 resin was partial throughout the adsorptive process; and with the HPA512L resin, complete adsorption occurred only after 2 h. In turn, the sugars (xylose and arabinose) and polyols (xylitol and arabitol) presented similar adsorption profiles, often with overlapping experimental data. Although there is no separation of polyols from residual sugars, this overlap can be mitigated in steps prior to the product purification process, that is, by optimizing fermentation conditions to increase polyol production while reducing residual sugar content.^6^

As we saw previously, acidic activated carbon was the adsorbent that most quickly clarified the model solution containing polyols and contaminants. This can be observed by the decrease in relative concentration values at 420 and 560 nm, commonly used to observe the color of sugar solutions. Activated carbon is a very versatile material and a cheaper raw material.^45^ Its principle of action may involve ion attraction when its surface is charged,^29^ but it is also a material with a highly porous structure, which is why it is widely used in decolorization and water treatment processes.^31,46^ These two effects were possibly what caused the clarification of the model solution in the first moments of adsorption.

In turn, the HPA512L resin is a strongly basic resin, based on trimethylammonium charged with the Cl^–^ ion.^41^ The clarification effect was possibly caused by the interaction with negatively charged species (e.g., proteins and phenolic compounds) in the aqueous medium, replacing the original counterion (Cl^–^).^33^ Therefore, the HPA512L resin clarified the medium with a lower rapidity than activated carbon, reaching complete clarification after 2.0 h of adsorption under the conditions evaluated.

Sepabeads SP700 resin is a synthetic, nonionic adsorbent composed of a copolymer of divinylbenzene and ethylvinylbenzene, characterized by a highly hydrophobic surface and absence of ionizable functional groups.^39^ Unlike HPA512L resin, whose adsorption mechanism is based on ion exchange, SP700 acts predominantly through hydrophobic interactions and van der Waals forces, favoring the retention of nonpolar or moderately polar compounds, such as phenolic pigments.^32,47^ In the experiments performed, SP700 promoted the removal of approximately 50% of the colored compounds (monitored by absorbance at 420 and 560 nm) in the initial contact times, maintaining this efficiency throughout the adsorption process.

It was observed that the adsorption kinetic profiles of polyols and interfering compounds presented similar behaviors at temperatures of 30 and 50 °C (Figures 4 and 5). However, at 50 °C, relevant particularities were noted. Under these conditions, the increase in temperature significantly favored the clarification effect promoted by the HPA512L resin. While the removal of pigmented compounds took approximately 2.0 h at 30 °C, at 50 °C, this effect occurred in the first minutes of adsorption (60 min), that is, the clarification effect was achieved in half the time. This unprecedented effect can be observed in Figures 3C and 4C and suggests an operational advantage when the HPA512L resin is used at high temperatures, making the process more efficient and potentially more economical in industrial applications. It is known that increasing the temperature increases the diffusion rate of the sorbate molecule through the external boundary layer and the internal pores of the adsorbent particle.^48^ Ferrah and collaborators^49^ observed that increasing the temperature increased the adsorption in an ionic process and that this may be due to the acceleration of some originally slow sorption steps or to the greater mobility of the ions from the solution to the functionalized surface of the resin. On the other hand, a reduction in the adsorption capacity of activated carbon was observed at 50 °C, possibly due to the weakening of the interaction forces between the adsorbate and the adsorbent at higher temperatures.^50^

**Figure 5:**
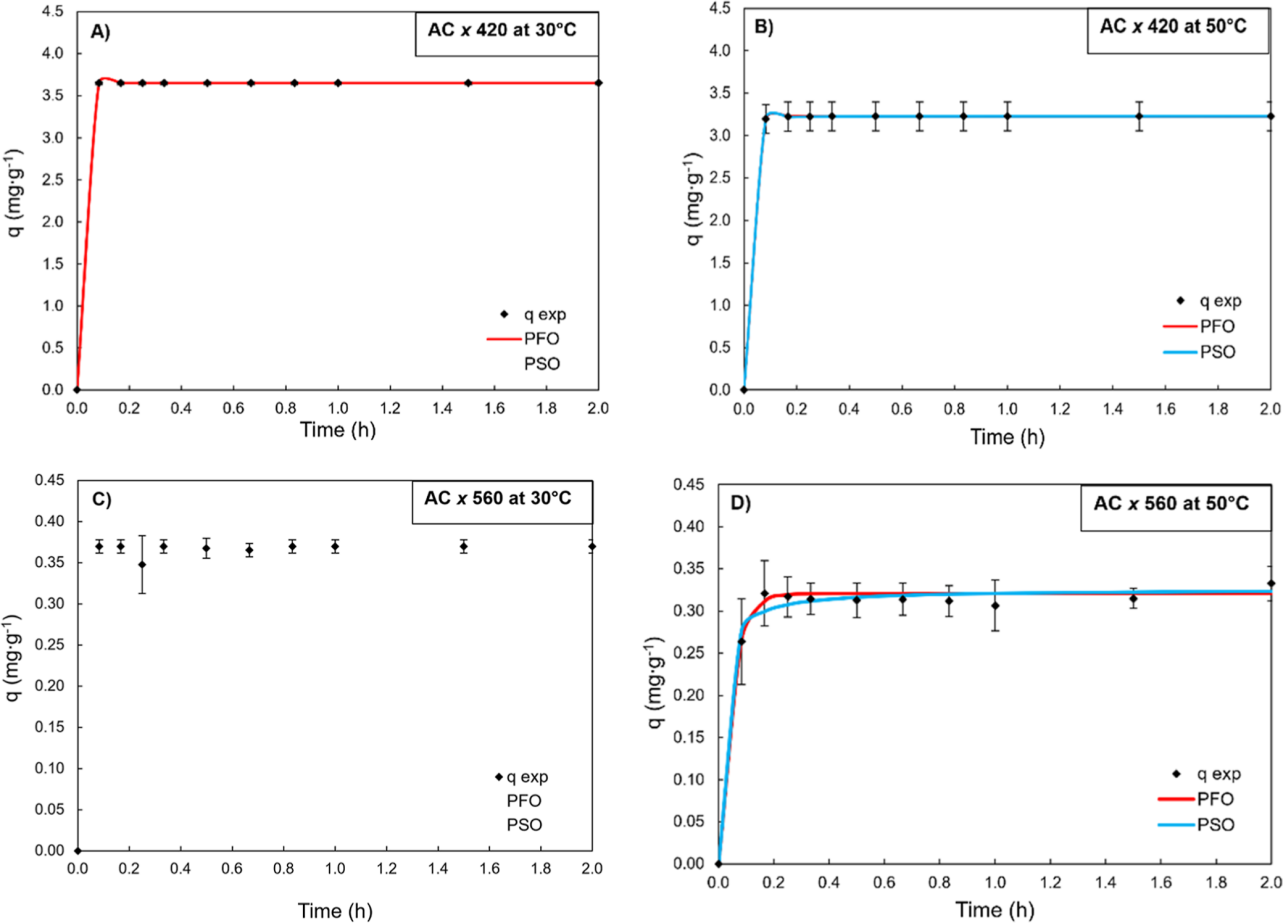
Fitting of PFO and PSO kinetic models to the adsorption curves of colored compounds at 420 nm (A,B) and 560 nm (C,D) on acid-activated (AC) carbon resin at 30 (A,C) and 50 °C (B,D).

The clarification observed with the use of acidic activated carbon and HPA512L ion-exchange resin can be attributed to the ability of these materials to interact with the functional groups present in alkaline lignin, used as a model for colored compounds. Lignin has a complex and heterogeneous structure, rich in phenylpropanoid units with free phenolic, carboxylic, methoxyl, and hydroxyl groups, which are partially ionized in an alkaline medium.^51^ This behavior can be justified by the lower affinity of the SP700 resin compared to acidic activated carbon and HPA512L resin, since the latter two present ionic functionality, acidic and basic, respectively, while SP700 is a neutral adsorbent, without charged groups that favor electrostatic interactions with the compounds present in the model solution.

In summary, activated carbon, with a high surface area and abundance of oxygenated groups on the surface (especially after acid activation), favors adsorption by π–π interactions, van der Waals forces, and hydrogen bonds.^29^ HPA512L resin, on the other hand, is a strongly basic type I ion-exchange material, functionalized with trimethylammonium groups (−N^+^(CH_3_)_3_) and supported on a highly porous styrene-divinylbenzene matrix.^41^ With a good specific surface area, pore volume, and average pore radius, this resin allows the diffusion of medium to high molecular weight molecules, such as soluble lignin fragments, promoting their removal through electrostatic interactions and ion-exchange mechanisms.^31^ These properties explain its efficient performance in clarifying the medium, especially at high temperatures. Therefore, the activated carbon adsorbents and the HPA512L resin stand out, as they obtained good separation of the interfering compounds and were able to clarify the medium without much damage to the polyols.

### Kinetics

The PFO and PSO models were fitted to the kinetic curves to evaluate both the performance and the adsorption mechanism involved in these processes. The fits for the polyols arabitol and xylitol and for the sugars xylose and arabinose are presented in Figures S2–S4 in the Supporting Information. The fitted parameters can also be seen in Tables S1–S4 for the kinetic models, which had a good fit, but PSO had the highest coefficient of determination and the lowest absolute deviation values of the experimental points.

It was chosen to present the results for the colored components here since it is of interest to understand the kinetic mechanisms for the purpose of clarifying the medium. Therefore, Figure 5 presents the kinetic data and the model fits for the acid activated carbon, Figure 6 for the SP700 resin, and Figure 7 for the HPA512L resin. The adjusted model parameters, the coefficients of determination, and the average absolute deviations are shown in Table 4.

**Figure 6:**
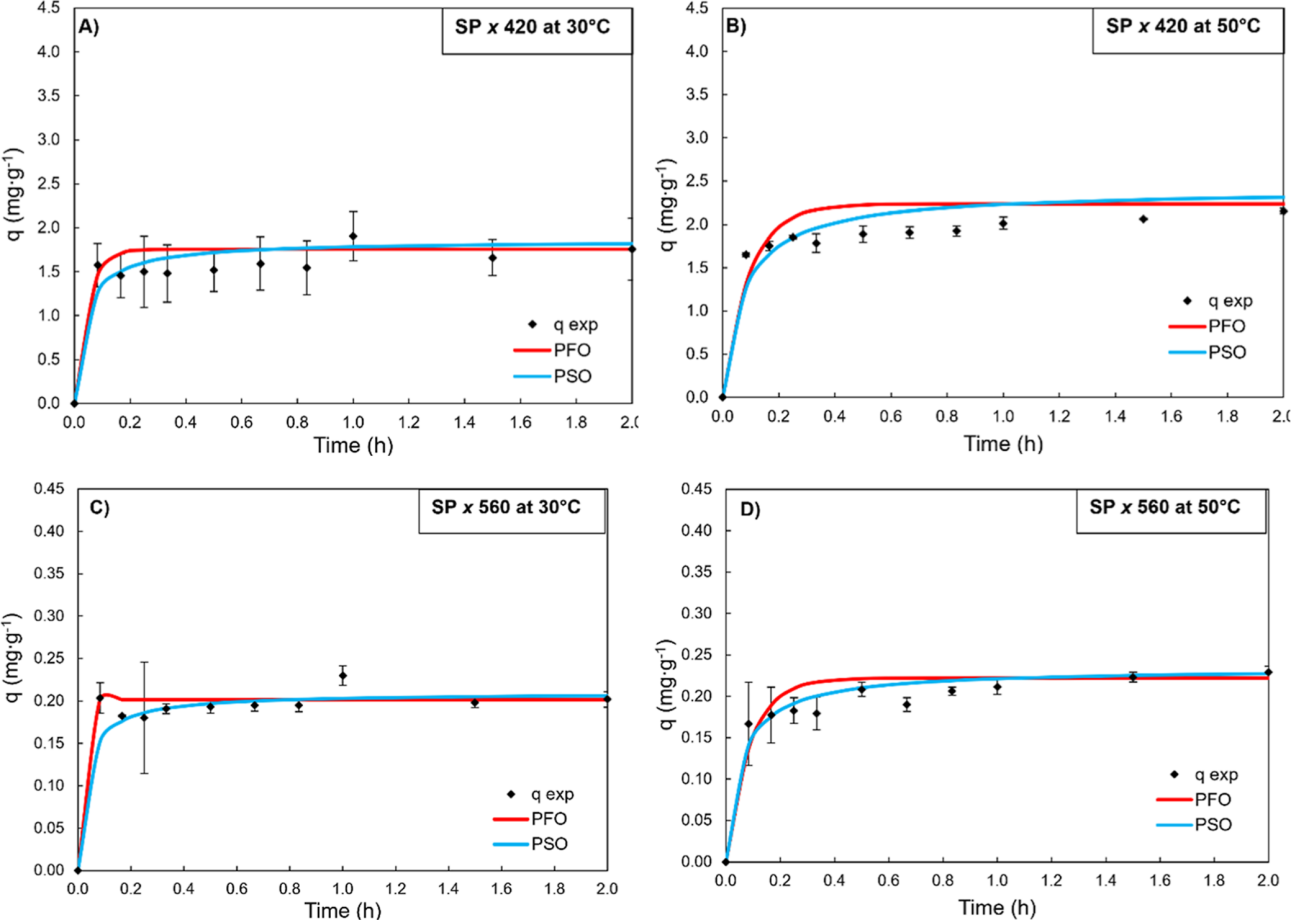
Fitting of PFO and PSO kinetic models to the adsorption curves of colored compounds at 420 nm (A,B) and 560 nm (C,D) on SP700 resin (SP) at 30 (A,C) and 50 °C (B,D).

**Figure 7:**
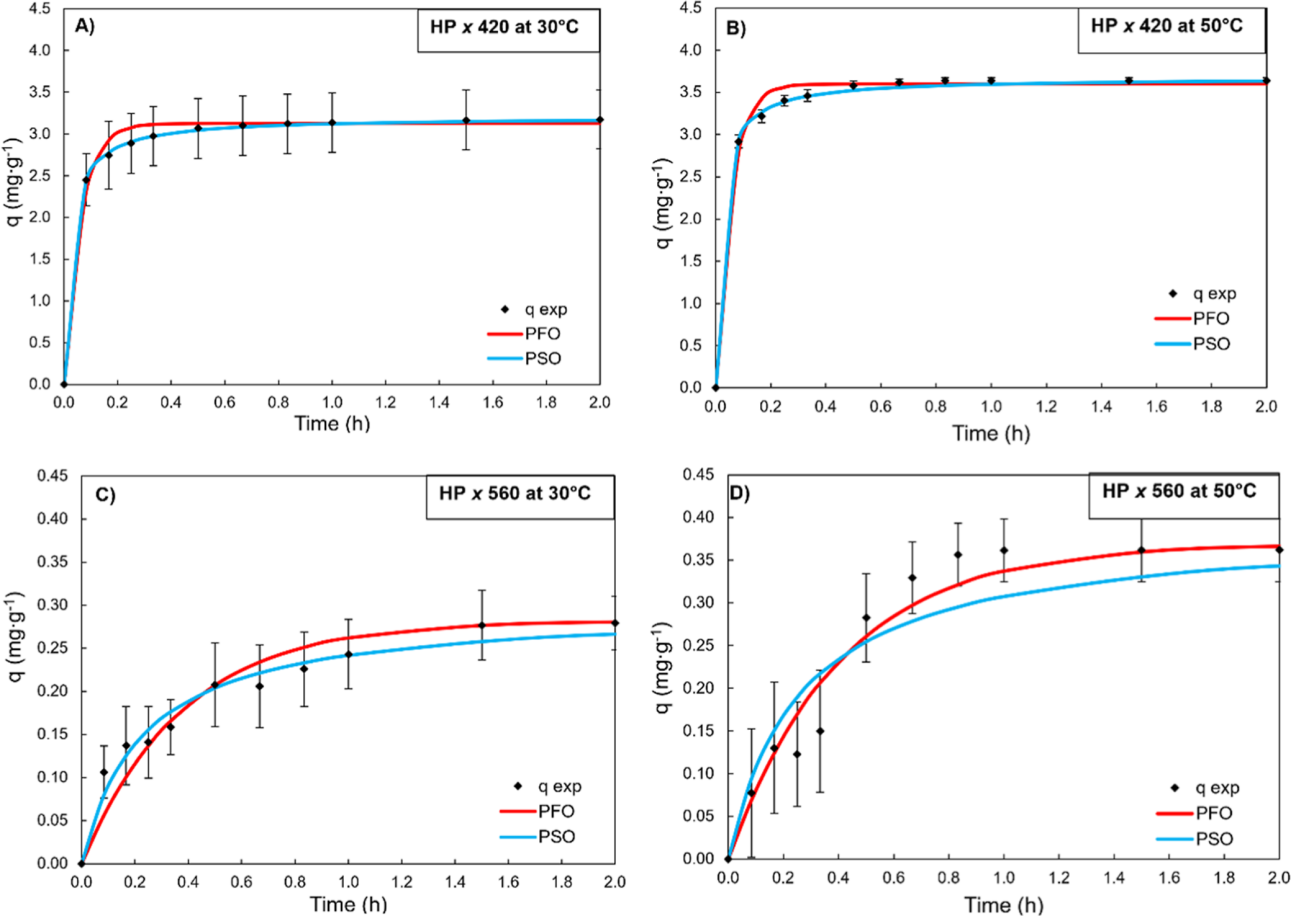
Fitting of PFO and PSO kinetic models to the adsorption curves of colored compounds at 420 nm (A,B) and 560 nm (C,D) on HPA512L resin (HP) at 30 (A,C) and 50 °C (B,D).

**Table 4:** Kinetic Model Parameters of Colored Compounds at 420 and 560 nm on Acid-Activated Carbon and SP700 and HPA512L Resins at 30 and 50 °C

			acid-activated carbon		resin SP700		resin HPA512L	
	model	parameters	420 nm	560 nm	420 nm	560 nm	420 nm	560 nm
temperature 30 °C	experimental data	*q*_e_ (mg·g^–1^)	3.652	0.368	1.696	0.199	2.878	0.207
	pseudo-first-order (PFO)	*q*_e_ (mg·g^–1^)	3.650	0.368	1.759	0.201	3.124	0.282
		*k*_1_ (h^–1^)	309.713	209.972	21.385	192.876	16.271	2.652
		*R* ^2^	>0.999	0.992	0.797	0.930	0.986	0.938
		%D	<0.001	0.048	1.414	0.435	0.003	3.332
	pseudo-second-order (PSO)	*q*_e_ (mg·g^–1^)	-	-	1.854	0.209	3.202	0.297
		*k*_2_ (g·mg^–1^·h^–1^)	-	-	13.842	151.082	12.109	14.732
		*R* ^2^	-	-	0.869	0.963	>0.999	0.975
		%D	-	-	0.759	1.375	0.003	0.738
temperature 50 °C	experimental data	*q*_e_ (mg·g^–1^)	3.225	0.315	2.056	0.212	3.527	0.294
	pseudo-first-order (PFO)	*q*_e_ (mg·g^–1^)	3.227	0.321	2.238	0.222	3.597	0.369
		*k*_1_ (h^–1^)	55.969	21.368	10.550	11.394	18.054	2.456
		*R* ^2^	>0.999	0.990	0.662	0.868	0.991	0.964
		%D	<0.001	0.068	1.882	0.503	<0.001	2.344
	pseudo-second-order (PSO)	*q*_e_ (mg·g^–1^)	3.230	0.326	2.398	0.233	3.672	0.388
		*k*_2_ (g·mg^–1^·h^–1^)	453.659	211.135	5.553	77.420	12.988	9.790
		*R* ^2^	>0.999	0.987	0.760	0.948	0.998	0.908
		%D	<0.001	0.091	1.046	0.144	0.015	5.889

Figure 5 presents the kinetic profiles of the adsorption capacity of the colored compounds read at 420 and 560 nm for acidic activated carbon at temperatures of 30 and 50 °C and the fitted pseudo-first-order and pseudo-second-order models. The results show that the maximum adsorption capacity at 50 °C for the compounds at 420 and 560 nm was slightly lower than the capacity at 30 °C. Jedli et al.^50^ also noted in their study that the adsorption capacity of activated carbon decreased with increasing temperature for a CO_2_ adsorption system. Therefore, this decrease in capacity with increasing temperature can be explained due to the binding forces between the adsorbate and the adsorbent decreasing with increasing temperature, leading to the decline in the adsorption capacity. The reduction in capacity with an increase in temperature is consistent with an exothermic adsorption process (negative heat of adsorption), which decreases overall affinity at higher temperatures.

The adsorption capacity of the colored compounds occurred extremely rapidly, especially at 420 nm, where absorbance values dropped to zero already at the second experimental time point of 5 min (*C*·*C*_0_^–1^ ∼ 0). This immediate removal made it difficult to properly fit the kinetic models under these conditions (Table 4), as the lack of intermediate data points between the initial and equilibrium stages limited the ability to mathematically describe the adsorption profile. Consequently, no reliable fits were obtained for the pseudo-first-order or pseudo-second-order models at 30 °C for this wavelength. This behavior suggests that adsorption is dominated by rapid surface interactions, likely driven by the high surface area and abundance of oxygenated functional groups in the acid-treated charcoal, which favor π–π interactions and strong affinity for phenolic and aromatic structures present in the solution.^29,45^ These findings highlight the remarkable ability of these types of carbons to promote the rapid and effective removal of colored compounds, although their kinetics may fall outside the typical assumptions of stepwise adsorption processes modeled by classical approaches.

The results of the kinetic profiles of the adsorption capacity for the SP700 resin and the models fitted to the experimental data are presented in Figure 6. The synthetic resin SP700 presented an adsorption capacity for the colored compounds at 420 and 560 nm that did not change much with the increase in temperature from 30 to 50 °C. The experimental values of *q*_e_ ranged from 1.5 to 2.0 mg·g^–1^ and 0.15 to 0.20 mg·g^–1^ for the 420 and 560 nm compounds, respectively, under the different conditions tested.

Despite the low adsorbed amounts compared to those of acidic carbon, the applied kinetic models fitted the data reasonably well. In particular, the pseudo-second-order (PSO) model performed better in most cases, with higher coefficients of determination (*R* ^2^), especially at 30 °C and 560 nm (*R* ^2^ = 0.963) and at 50 °C and 560 nm (*R* ^2^ = 0.948), as well as low percentage deviations (<1.37%). These results suggest that the adsorption kinetics of colored compounds on SP700 resin is better described by the PSO model, indicating a possible control by specific chemical interactions between the compounds and the active sites of the resin.

The PFO and PSO models were satisfactorily fitted to the experimental kinetic data (Figure 7 and Table 4) for the colored compounds at 420 and 560 nm on the HPA512L resin at 30 and 50 °C. For the compounds at 420 nm and 30 °C, the best-fitting model was PSO, with an *R* ^2^ of >0.999 and a mean absolute deviation of <0.00%. At 50 °C for 420 nm, the PFO and PSO models showed good agreement with the experimental data, with *R* ^2^ values of 0.991 and 0.998 and mean absolute deviations of <0.000% and 0.015%, respectively.

These results are illustrated in Figure 7, which presents the experimental data points together with the PFO and PSO curves fitted based on the kinetic parameters summarized in Table 4. The superior performance of the PSO model, particularly at 30 °C, suggests that the adsorption mechanism on HPA512L is predominantly governed by chemisorption, involving specific interactions between the functional groups of the colored compounds and the quaternary ammonium groups present in the resin. These interactions may include electrostatic attraction, hydrogen bonding, and even ion-exchange phenomena, especially considering the partial ionization of phenolic moieties in lignin under the experimental conditions. The porous structure of the resin and its high-water content may also facilitate molecular diffusion and surface accessibility, contributing to the rapid and efficient removal of colored compounds. The observed kinetic behavior reinforces the potential of HPA512L for the clarification of complex fermentation broths, where selective and strong adsorbent–solute interactions are essential for effective purification. These findings reinforce that the adsorption kinetics on HPA512L are governed by chemisorption mechanisms, as described by the pseudo-second-order model,^27^ which has been shown to outperform the pseudo-first-order model originally proposed in ref^26^ in systems where specific interactions dominate the adsorption process.

Kinetic modeling revealed that the adsorption of colored compounds at 420 and 560 nm fitted well to the pseudo-first-order and pseudo-second-order models, depending on the adsorbent. Acid-activated carbon exhibited extremely fast adsorption kinetics, with high *k*_1_ values and almost instantaneous removal at early time points, which is characteristic of systems governed by physisorption and surface diffusion. The *k*_1_ values for colored compounds at 420 and 560 nm at 30 °C ranged from 5.16 min^–1^ to 3.5 min^–1^, which corroborates the claim that it clarified the solution within the first few minutes of adsorption. Similarly, a similar result is found in ref 52 where he used activated carbon to adsorb a dye from a medium, but its values were higher than those found in this work, possibly due to its single-component system.

In contrast, HPA512L resin exhibited better fits with the pseudo-second-order model (*R* ^2^ > 0.999 at 420 nm), indicating that chemisorption may play a role in the interaction between resin and contaminants. The values of maximum adsorption capacities predicted by the models were similar to those observed experimentally, and the kinetic constants were lower (2.4–192.9) than those of activated carbon (21.4–309.7). Similar results were found for the HP20-like Dowex S112 resin, from the same family as HPA512L.^53^ Note that the *k* values in this work are in hours. Finally, SP700 resin showed slower kinetics and lower *R* ^2^ values, suggesting limited interaction or diffusion resistance. The same range of *k*_1_ values (0.06–0.13 h^–1^) were found in the work of Steffes et al.,^32^ who used the SP70 resin to recover β-carotene.

### Adsorption Isotherms

The experimental data for all components were fitted to the isotherm models mentioned in this work. However, satisfactory fits were not obtained for the colored compounds. Their adsorption curves are presented in Supporting Information (Figures S5 and S6) and, although experimental data are available for all components (as shown in Figure 8), isothermal modeling focused on polyols. This choice was due to operational limitations that prevented obtaining additional data at lower concentrations for the colored compounds, which compromised the reliable fitting of their isotherms. On the other hand, the modeling of the polyols was essential to investigate the selective adsorption behavior of the materials, allowing evaluation of both the potential for contaminant removal and the possibility of separation between structurally similar compounds, such as xylitol and arabitol, which are isomers. To this end, equilibrium studies were conducted in multicomponent systems using acidic activated carbon and HPA512L resin, at temperatures of 30 and 50 °C. The model solution containing the polyols was used to simulate a fermented broth, and the results obtained are presented in Figure 8.

**Figure 8:**
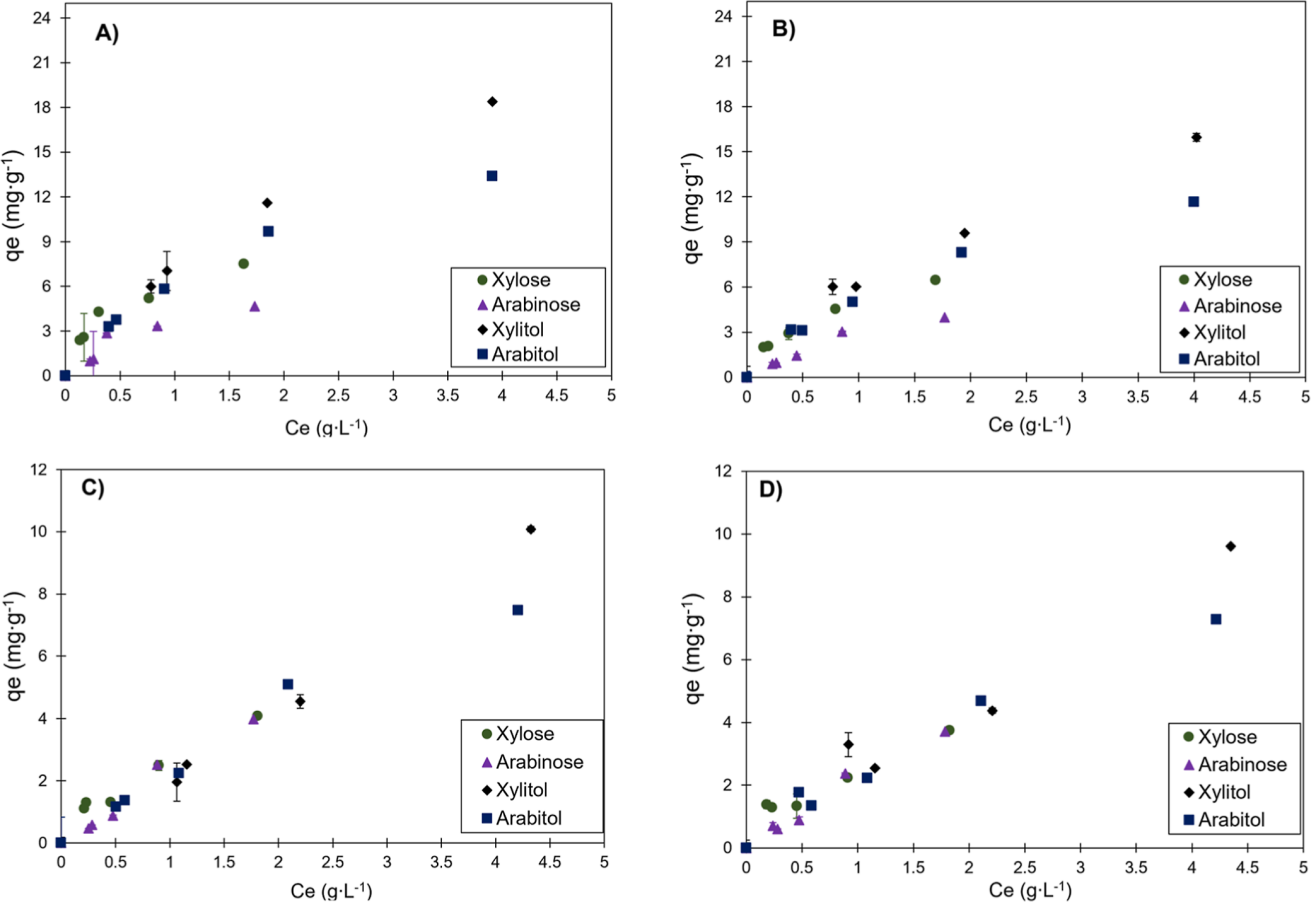
Adsorption isotherms of the model solution: on acidic activated carbon at 30 °C (A), on acidic activated carbon at 50 °C (B), on HPA512L resin at 30 °C (C), and on HPA512L resin at 50 °C (D). Adsorbent content of 50 g L^–1^, solution volume of 0.01 L.

The results in Figure 8 do not show differences in the behavior of the components of the model solution with increasing temperature. Both components show similar isotherm behavior at equilibrium, showing favorable behavior. The acidified activated carbon showed favorable isotherms, the adsorption capacity of which for xylitol was 18 mg·g^–1^ and for arabitol was 12 mg·g^–1^. However, a difference is noted between the two adsorbents analyzed. The HPA512L resin showed an adsorption capacity approximately half that of the activated carbon, reaching approximately 10 mg·g^–1^ for xylitol and 7.5 mg·g^–1^ for arabitol. This behavior suggests the predominance of specific interactions, increasing its adsorption capacity against the HPA512L resin.^29^

The HPA resin, in turn, presented isotherms with an intermediate profile between favorable and linear, indicating less specific adsorption mechanisms and lower sensitivity to the polyols and sugars evaluated. The results demonstrate that the microstructure and surface chemistry of the acidified activated carbon provide greater efficiency in the selective removal of these interferents, especially for molecules of smaller molecular size, while the HPA resin may present advantages in systems with a more complex composition. These insights are particularly relevant for the development of purification processes for streams containing biomass byproducts.

Based on the observed performance of the adsorbents in the previous steps, adsorption equilibrium studies were conducted to determine the maximum adsorption capacity of the polyols present in the model solution, using acid activated carbon and HPA512L resin at temperatures of 30 and 50 °C. The experimental data were fitted to different isothermal models, including Extended Langmuir (EL), Competitively Modified Langmuir, Noncompetitive Langmuir, and Langmuir–Freundlich (LF), in order to identify the one that best described the adsorptive behavior of the system (Table 5).

**Table 5:** Extended and Modified Langmuir Isotherm Adsorption Constants for Xylitol and Arabitol Polyols on Acidic Activated Carbon and HPA512L Resins at 30 and 50 °C

			acid-activated carbon	resin HPA
		parameters	xylitol–arabitol	xylitol–arabitol
T30	Extended Langmuir	*q*_max1_ (g·g^–1^)	0.017	25.917
		K_1_	0.512	<0.001
		K_2_	–0.600	0.092
		*R* ^2^	0.999	0.991
		%D	0.494	12.475
	modified competitive Langmuir	*q*_max_ (g·g^–1^)	0.016	41.924
		K_1_	0.588	>0.001
		K_2_	–0.610	0.350
		*R* ^2^	0.999	0.997
		%D	0.659	47.602
T50	extended Langmuir	*q*_max1_ (g·g^–1^)	0.053	25.171
		K_1_	0.152	>0.001
		K_2_	0.200	0.138
		*R* ^2^	0.997	0.989
		%D	1.304	0.060
	modified competitive Langmuir	*q*_max_ (g·g^–1^)	0.053	26.000
		K_1_	0.153	>0.001
		K_2_	0.242	0.300
		*R* ^2^	0.997	0.989
		%D	2.404	14.774

Figure 9 presents the experimental data and the values predicted by the models that demonstrated a good fit to the multicomponent system. Although all models described in the methods section were tested, only the Extended Langmuir (EL) and Competitively Modified Langmuir isotherms presented satisfactory performance, being able to adequately represent the adsorption equilibrium of the evaluated compounds. The *R* ^2^ values obtained (Table 5) indicate that the Extended Langmuir (EL) and Competitively Modified Langmuir (MCL) models presented a good fit to the experimental data of xylitol and arabitol adsorption on acid-activated carbon and HPA512L resin, suggesting that these models are mathematically adequate to describe the behavior of the system. However, the occurrence of negative parameters in some fits, such as K_2_ values, especially for the resin, indicates limitations in the physicochemical interpretation of the results.^18^ This behavior may be related to the complexity of the multicomponent system prepared from synthetic hemicellulose hydrolysate, in which the specific interactions between the solutes and the adsorbents may not be fully represented by the assumptions of the models used. Thus, although the fits presented high correlation coefficients, the physical validity of the parameters should be carefully considered in interpreting the results.

**Figure 9:**
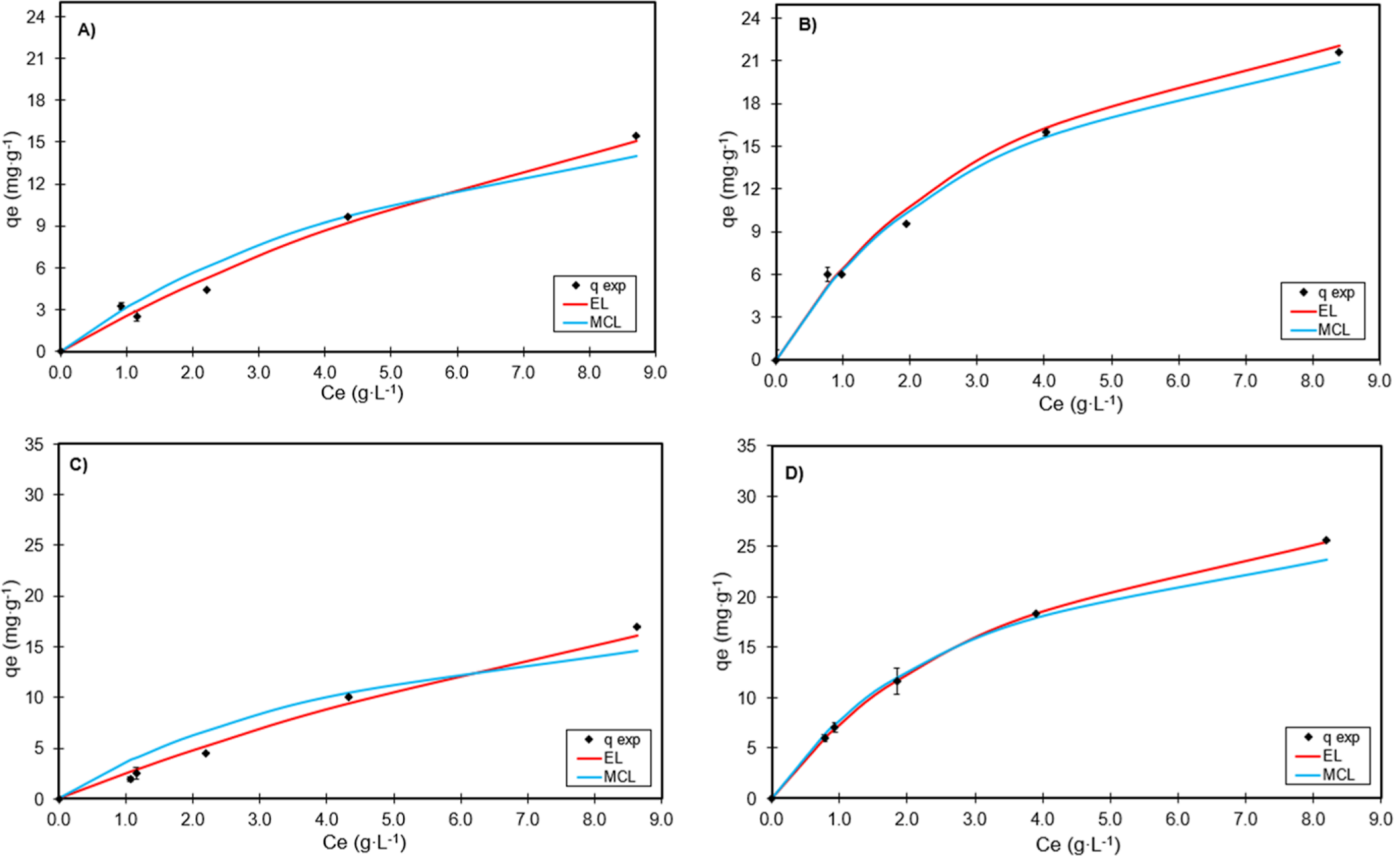
Fitting the models to the experimental data for the polyols: in activated carbon at 30 °C (A), in activated carbon at 50 °C (B), in HPA512L resin at 30 °C (C), and in HPA512L resin at 50 °C (D).

Furthermore, the strong fit of the Extended Langmuir (EL) and Modified Competitive Langmuir (MCL) models to the experimental data supports the hypothesis of direct competition between polyols for shared adsorption sites.^26^ The EL model assumes that all adsorbates compete uniformly for the same type of site, and its good performance (*R* ^2^ > 0.99 and %D < 1.5% in most cases) confirms that surface homogeneity and monolayer adsorption are reasonable assumptions for both adsorbents, particularly activated carbon.

The MCL model, while slightly more complex, includes interaction parameters that offer a more refined interpretation of the competitive behavior between solutes. Its superior fit for HPA512L at higher temperatures suggests that additional physicochemical effects—such as differences in solute affinity or partial site blocking—may influence multicomponent adsorption. The relatively low deviations (%D < 2.5%) and consistent trends in *q*_max_ values support the reliability and added interpretive value of the MCL model. Although the interaction of polyols with different adsorbents is novel, Jiang’s et al.^54^ work studied the interaction with activated carbon and mannitol and obtained results close to the parameters found in this one.

Together, these findings demonstrate that both the EL and MCL models are not only statistically robust but also physically meaningful within the studied system, effectively capturing the competitive adsorption dynamics between arabitol and xylitol. In future applications, these models could support the optimization of adsorbent loading and flow conditions in continuous systems by providing predictive insight into the capacity and selectivity under competitive scenarios.

## Conclusions

This study identified acid-activated carbon and HPA512L ion-exchange resin as effective adsorbents for selectively removing phenolic and chromophore compounds from polyol-rich fermented broths while minimizing the loss of target polyols, arabitol, and xylitol. Acid-activated carbon showed higher adsorption capacity due to its surface functionalization and microporous structure, enabling rapid and efficient clarification. HPA512L resin, in turn, exhibited improved performance at elevated temperatures, suggesting enhanced diffusion and electrostatic interactions via its quaternary ammonium groups. Kinetic modeling confirmed that adsorption followed pseudo-second-order behavior, consistent with chemisorption mechanisms. The applicability of the Extended and Modified Langmuir isotherms confirmed competitive adsorption between solutes. Importantly, the adsorbents maintained a stable performance across a broad pH range, simplifying process integration. Overall, this work advances the development of a scalable, selective, and sustainable downstream purification strategy for biotechnologically produced polyols from agroindustrial residues. Future research should focus on fixed-bed column operation and regeneration strategies to support industrial viability.

## Supplementary Material



## Notes

Schematic of the work, kinetics, modeling, and kinetic parameters for sugars and polyols and isotherms, modeling, and isothermal parameters for the components at 420 and 560 nm (PDF)
