# Supplementary material for: Selective Removal of Contaminant Compounds from Polyol-Rich Fermented Broth by Multicomponent Adsorption on New Adsorbent Materials
Source: ACS Omega. 2025 Nov 27;10(48):59535–51. doi: 10.1021/acsomega.5c09201 (PMC12771261; doi:10.1021/acsomega.5c09201)
Supplement: Supplementary file 1 [file ao5c09201_si_001.pdf]

Supporting information

Selective removal of contaminant compounds from polyol-rich fermented broth by multicomponent adsorption on new adsorbent materials

Danielle Garcia Ribeiro Galvão*, Jan Galvão Gomes; Maria Eduarda Rampin de Almeida; Marcus Bruno Soares Forte[1]

Bioprocess and Metabolic Engineering Laboratory (LEMeB), Food Engineering and Technology Department (DETA), Faculty of Food Engineering (FEA), University of Campinas (UNICAMP), R. Monteiro Lobato, 80, Campinas, São Paulo, 13083-862, Brazil.

*forte@unicamp.br/ danisgalvao@outlook.com

Figure S1 presents the work schematic. It presents the work steps in a simplified format, as well as which step uses which matrix, between real broth and model solution.

Figure S1 - Figure S1 presents the work scheme.

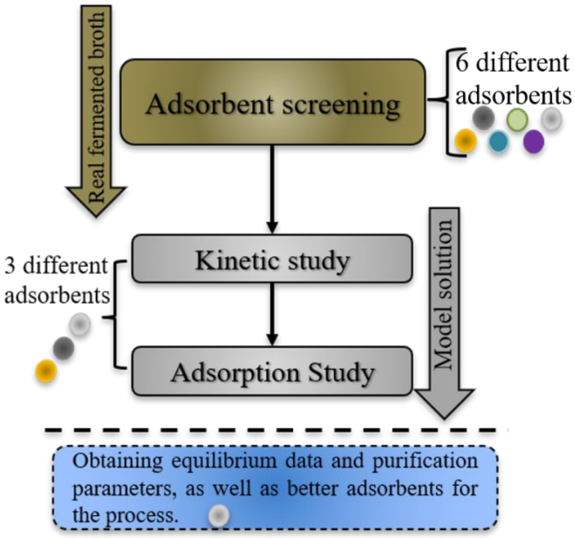

Figures S2, S3 and S4 show the experimental data and the adjusted kinetic models of each component of the model solution at temperatures of 30 and 50 °C in the different adsorbents.

Figure S2 - The experimental data and the fitted kinetic models of each component of the model solution at temperatures of 30 and 50 °C in Acid Activated Carbon (AC).

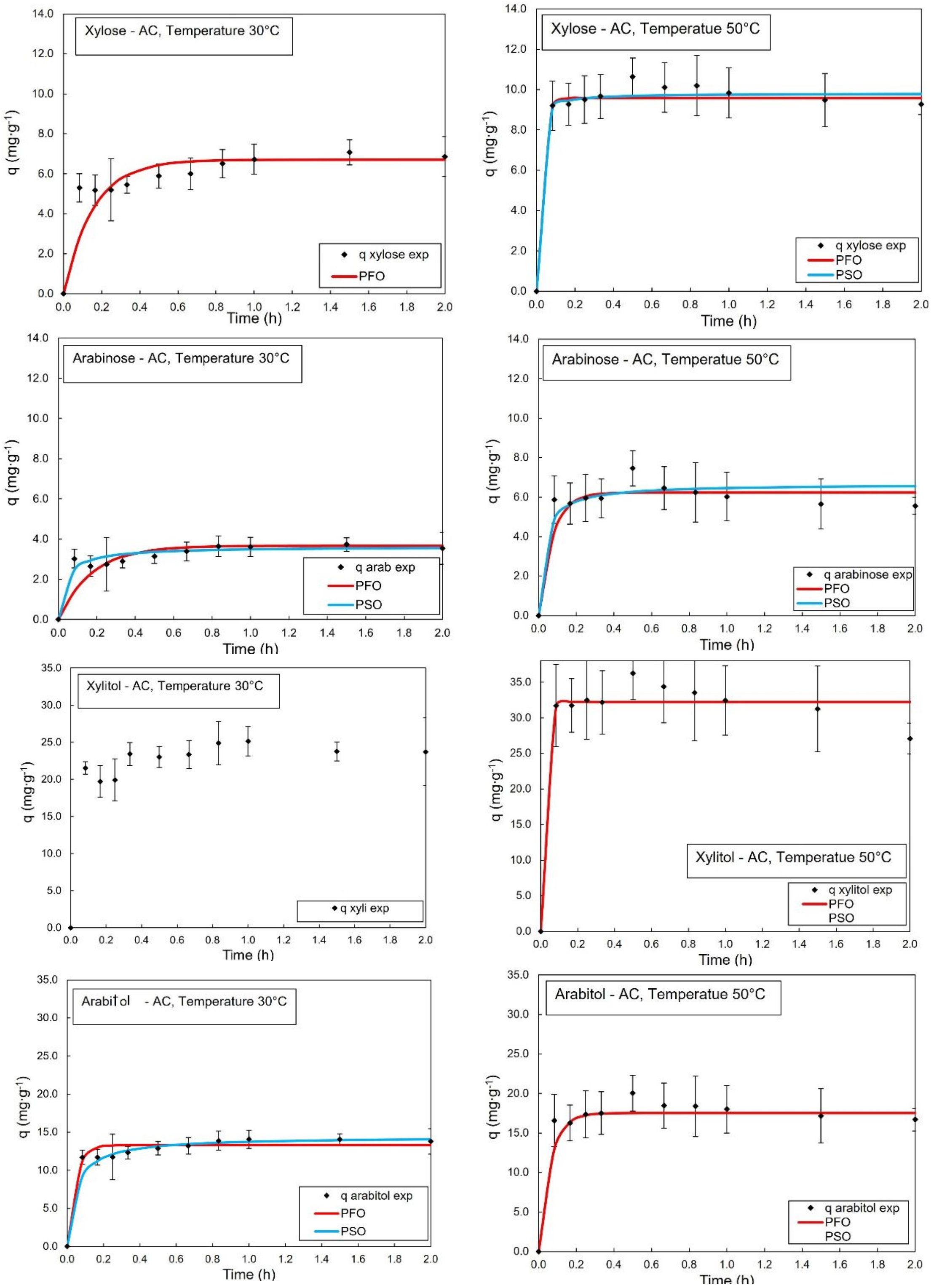

Figure S3 - The experimental data and the fitted kinetic models of each component of the model solution at temperatures of 30 and 50 °C in SP700 resin (SP).

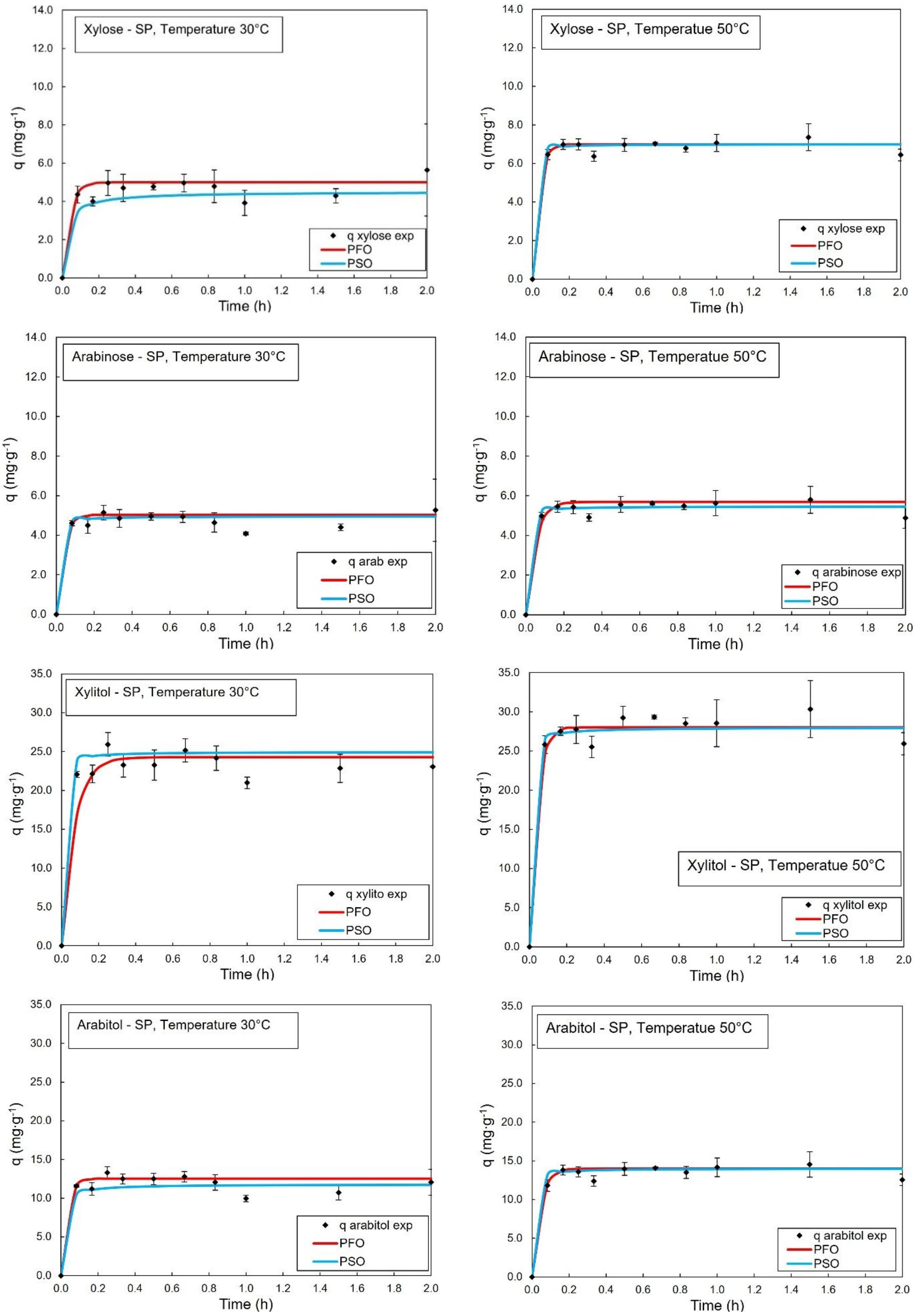
Xylose - SP, Temperature 30°C
Xylose - SP, Temperatue 50°C
Arabinose - SP, Temperature 30°C
Arabinose - SP, Temperatue 50°C
Xylitol - SP, Temperature 30°C
Xylitol - SP, Temperatue 50°C
Arabitol - SP, Temperature 30°C
Arabitol - SP, Temperatue 50°C
q xylose exp
q arab exp
q arabinose exp
q xylito exp
q xylitol exp
q arabitol exp
PFO
PSO
q (mg·g⁻¹)
Time (h)

Figure S4 - The experimental data and the fitted kinetic models of each component of the model solution at temperatures of 30 and 50 °C in HPA512L resin (HP).

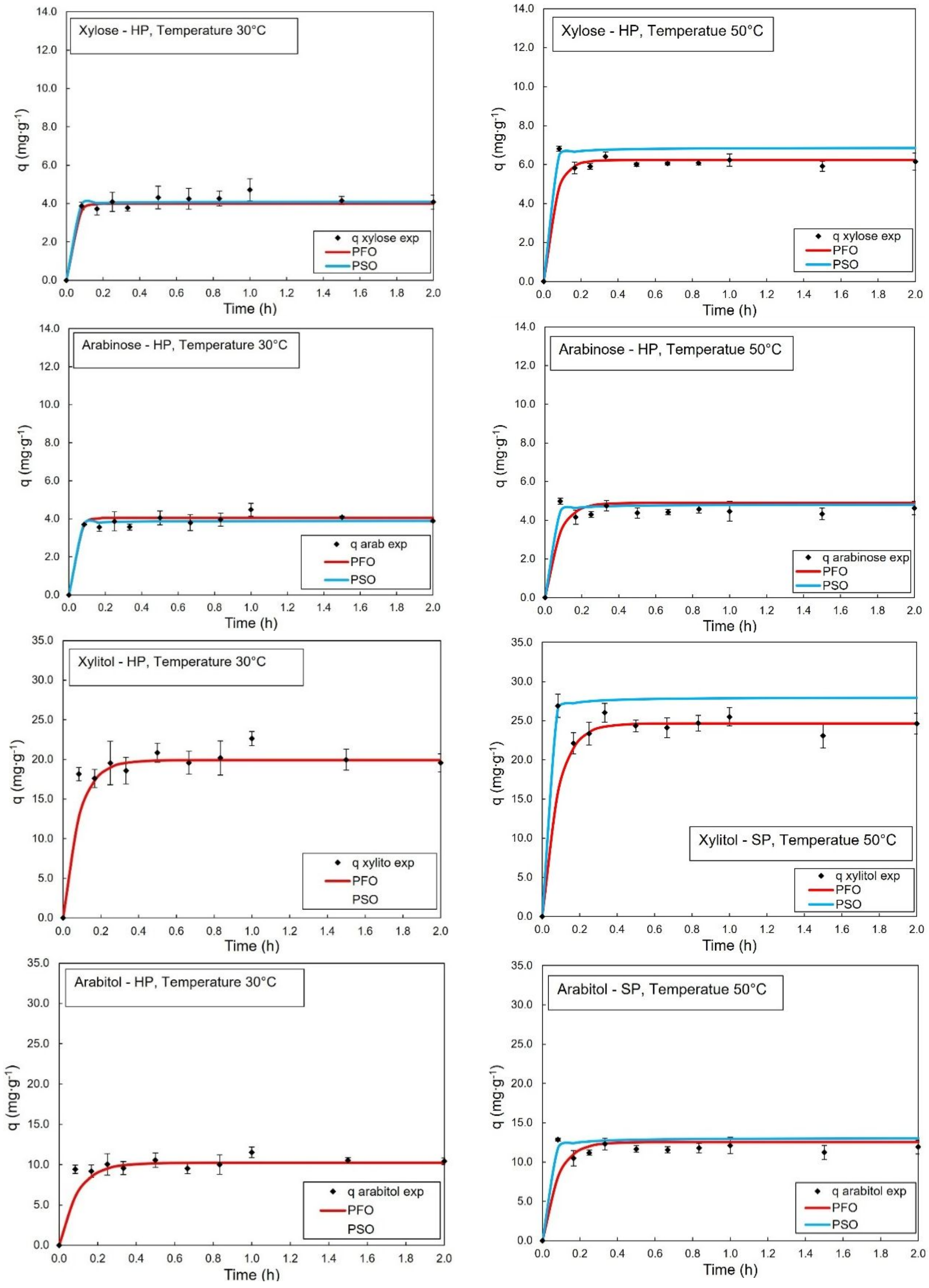

Tables S1, S2 and S3 present the kinetic parameters for each component of the model solution in the different adsorbents at temperatures of 30 and 50 °C.

Table S1 - Kinetic Model Parameters for each component of the model solution on acid activated carbon at 30 and 50 °C.

	Model	Parameters	Acid Activated Carbon			
			**Xylose**	**Arabinose**	**Xylitol**	**Arabinose**
**Temperature 30 °C**	Experimental data	q_e (mg·g^{-1})	5.871	3.107	22.626	12.447
	Pseudo-first-order (PFO)	q_e (mg·g^{-1})	6.702	3.660	-	13.306
		k_1 (h^{-1})	6.460	5.730	-	22.075
		R^2	0.938	0.955	-	0.943
		% D	4.18	2.33	-	2.31
	Pseudo-second-order (PSO)	q_e (mg·g^{-1})	-	3.625	-	14.412
		k_2 (g·mg^{-1}·h^{-1})	-	7.014	-	1.459
		R^2	-	0.901	-	0.995
		% D	-	0.610	-	1.590
**Temperature 50 °C**	Experimental data	q_e (mg·g^{-1})	9.660	5.995	32.024	17.554
	Pseudo-first-order (PFO)	q_e (mg·g^{-1})	9.581	6.235	32.216	17.517
		k_1 (h^{-1})	37.855	13.672	49.171	16.152
		R^2	0.998	0.996	>0.999	0.988
		% D	0.380	4.530	2.400	0.930
	Pseudo-second-order (PSO)	q_e (mg·g^{-1})	9.807	6.664	-	-
		k_2 (g·mg^{-1}·h^{-1})	15.918	4.884	-	-
		R^2	0.999	0.999	-	-
		% D	1.750	9.220	-	-

Table S2 - Kinetic Model Parameters for each component of the model solution on SP700 resin at 30 and 50 °C.

	Model	Parameters	SP700 Resin			
			**Xylose**	**Arabinose**	**Xylose**	**Arabinose**
**Temperature 30 °C**	Experimental data	q_e (mg·g^{-1})	4.646	4.415	21.847	10.887
	Pseudo-first-order (PFO)	q_e (mg·g^{-1})	5.000	5.024	24.264	12.540
		k_1 (h^{-1})	24.888	27.653	14.277	30.387
		R^2	0.981	0.990	0.994	>0.999
		% D	7.580	6.590	0.820	5.730
	Pseudo-second-order (PSO)	q_e (mg·g^{-1})	4.500	4.950	24.956	11.780
		k_2 (g·mg^{-1}·h^{-1})	8.278	43.101	10.618	7.761
		R^2	0.996	0.989	0.995	0.999
		% D	4.910	4.790	7.330	1.100
**Temperature 50**	Experimental data	q_e (mg·g^{-1})	6.767	5.313	27.595	13.290
		q_e (mg·g^{-1})	7.000	5.684	28.000	13.985

	Pseudo-first-order (PFO)	k_1 (h^{-1})	29.228	20.428	25.533	23.045
		R^2	0.998	0.997	0.994	0.983
		% D	4.090	8.190	8.410	7.540
	Pseudo-second-order (PSO)	q_e ($mg \cdot g^{-1}$)	7.000	5.462	28.000	14.000
		k_2 ($g \cdot mg^{-1} \cdot h^{-1}$)	51.810	46.325	7.462	13.252
		R^2	0.998	0.999	0.999	0.997
		% D	5.330	10.100	6.610	7.180

Table S3 - Kinetic Model Parameters for each component of the model solution on HPA512L resin at 30 and 50 °C.

			**HPA512L Resin**			
	**Model**	**Parameters**	**Xylose**	**Arabinose**	**Xylose**	**Arabinose**
**Temperature 30 °C**	Experimental data	q_e ($mg \cdot g^{-1}$)	3.811	3.655	18.053	9.406
	Pseudo-first-order (PFO)	q_e ($mg \cdot g^{-1}$)	4.000	4.056	19.896	10.234
		k_1 (h^{-1})	28.238	28.667	12.173	10.645
		R^2	0.994	0.993	0.996	0.997
		% D	4.430	5.130	0.730	0.890
	Pseudo-second-order (PSO)	q_e ($mg \cdot g^{-1}$)	4.102	3.890	-	-
		k_2 ($g \cdot mg^{-1} \cdot h^{-1}$)	83.850	69.005	-	-
		R^2	>0.999	0.997	-	-
		% D	2.850	0.730	-	-
**Temperature 50 °C**	Experimental data	q_e ($mg \cdot g^{-1}$)	6.205	4.510	24.643	11.801
	Pseudo-first-order (PFO)	q_e ($mg \cdot g^{-1}$)	6.236	4.895	24.635	12.547
		k_1 (h^{-1})	17.798	14.073	12.286	12.616
		R^2	0.998	0.991	0.995	0.990
		% D	1.720	9.620	8.380	8.460
	Pseudo-second-order (PSO)	q_e ($mg \cdot g^{-1}$)	6.870	4.823	25.014	13.080
		k_2 ($g \cdot mg^{-1} \cdot h^{-1}$)	27.878	30.724	4.304	8.669
		R^2	0.996	0.997	0.999	0.998
		% D	4.410	0.440	0.110	0.280

Figure S5 - Experimental data and fitted isotherm models for the component at 420 nm at temperatures of 30 and 50 °C in HPA512L (HP) resin and Acid activated carbon (AC).

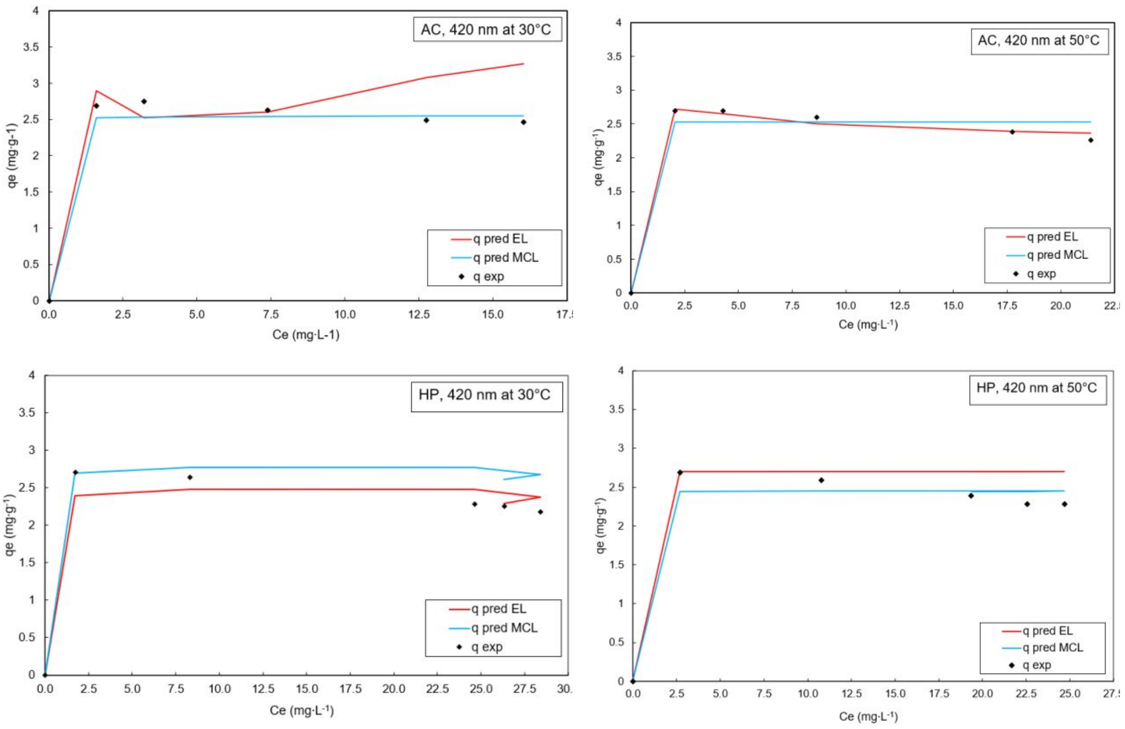

Figure S6 - Experimental data and fitted isotherm models for the component at 560 nm at temperatures of 30 and 50 °C in HPA512L (HP) resin and Acid activated carbon (AC).

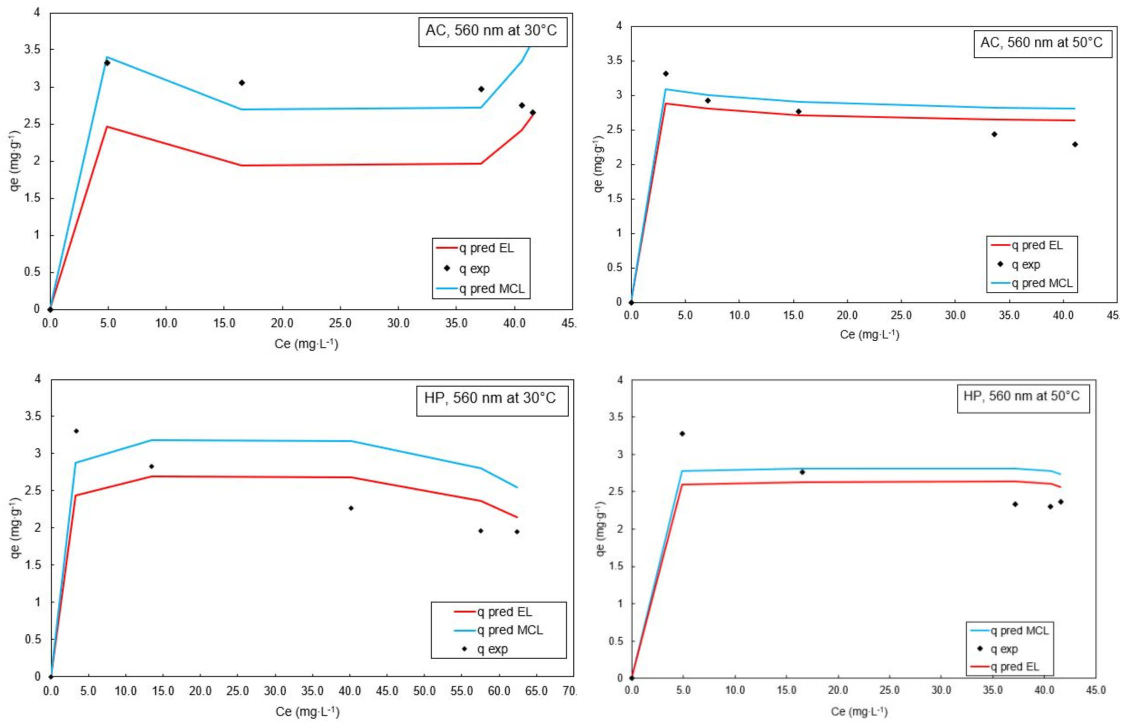

Table S4 - Kinetic Model Parameters for 420 and 560 nm components of the model solution on different adsorbents at 30 and 50 °C.

			Acid Activated Carbon	Resin HPA
		Parameters	420 nm – 560 nm	420 nm – 560 nm
T30	Extended Langmuir	q_{max1} ($g{\cdot}g^{-1}$)	4.50	4.0
		K_1	5.00	972703.1
		K_2	0.70	202172.9
		R^2	0.99	0.97
		%D	11.08	2.24
	Modified Competitive Langmuir	q_{max} ($g{\cdot}g^{-1}$)	2.55	5.5
		K_1	65.00	972703.0
		K_2	0.01	200000.0
		R^2	0.96	0.93
		%D	10.90	12.97
T50	Extended Langmuir	q_{max1} ($g{\cdot}g^{-1}$)	33.66	4.9
		K_1	0.77	374529.0
		K_2	5.27	172235.5
		R^2	0.994	0.970
		%D	21.87	8.20
	Modified Competitive Langmuir	q_{max} ($g{\cdot}g^{-1}$)	2.53	5.5
		K_1	2117136.94	200000.0
		K_2	2.60	100000.0
		R^2	0.89	0.82
		%D	3.53	22.40
